# Pre-existing H4K16ac levels in euchromatin drive DNA repair by homologous recombination in S-phase

**DOI:** 10.1038/s42003-019-0498-z

**Published:** 2019-07-05

**Authors:** Nobuo Horikoshi, Dharmendra Sharma, Fransisca Leonard, Raj K. Pandita, Vijaya K. Charaka, Shashank Hambarde, Nobuko T. Horikoshi, Puja Gaur Khaitan, Sharmistha Chakraborty, Jacques Cote, Biana Godin, Clayton R. Hunt, Tej K. Pandita

**Affiliations:** 1 Department of Radiation Oncology, The Houston Methodist Research Institute, Houston, TX 77030 USA; 2 Department of Nanomedicine, The Houston Methodist Research Institute, Houston, TX 77030 USA; 3 Department of Surgery, The Houston Methodist Research Institute, Houston, TX 77030 USA; 4 St. Patrick Research Group in Basic Oncology, Laval University Cancer Research Center, Quebec City, QC G1V4G2 Canada; 5 Present Address: Department of Radiation Oncology, Northwestern University Feinberg School of Medicine, Chicago, IL 60611 USA; 6 Present Address: Department of Surgery, Medstar Washington Hospital Center, Washington, DC 20010 USA

**Keywords:** Homologous recombination, DNA damage response

## Abstract

The homologous recombination (HR) repair pathway maintains genetic integrity after DNA double-strand break (DSB) damage and is particularly crucial for maintaining fidelity of expressed genes. Histone H4 acetylation on lysine 16 (H4K16ac) is associated with transcription, but how pre-existing H4K16ac directly affects DSB repair is not known. To answer this question, we used CRISPR/Cas9 technology to introduce I-SceI sites, or repair pathway reporter cassettes, at defined locations within gene-rich (high H4K16ac/euchromatin) and gene-poor (low H4K16ac/heterochromatin) regions. The frequency of DSB repair by HR is higher in gene-rich regions. Interestingly, artificially targeting H4K16ac at specific locations using gRNA/dCas9-MOF increases HR frequency in euchromatin. Finally, inhibition/depletion of RNA polymerase II or Cockayne syndrome B protein leads to decreased recruitment of HR factors at DSBs. These results indicate that the pre-existing H4K16ac status at specific locations directly influences the repair of local DNA breaks, favoring HR in part through the transcription machinery.

## Abstract (web summary)

Nobuo Horikoshi et al. report the use of CRISPR/Cas9 to insert cleavage and repair sites in gene-rich and gene-poor regions in human cell lines. They show that the frequency of double-stranded break repair by homologous recombination is higher in gene-rich regions with high levels of H4K16ac marks.

## Introduction

In order to maintain genomic stability in gene-rich regions, especially where transcription occurs consistently, high-fidelity DNA repair by HR is required to avoid deleterious mutations. Whether such high-fidelity DNA repair is dependent upon specific chromatin modifications is under studied due to technical reasons. The chromatin modification of histone H4 by acetylation at K16, H4K16ac^1^, carried out by the MOF (males absent on the first) acetyltransferase^2,3^, is associated with transcription^4–6^ and has been shown to alter chromatin structure^7^. We previously reported that depletion of MOF decreased H4K16ac and overall DNA DSB repair levels^2^, but how the modification impacts DSB repair and fidelity in structurally or functionally distinct regions, is not known. The restoration of DNA fidelity post-DSB induction is ensured by homology directed repair, specifically the HR pathway. Transcriptionally active regions reportedly recruit factors involved in HR-mediated DSB repair^8^, consistent with recent studies identifying DSB-induced histone modifications involved in preferential repair by HR^9^ and human C-NHEJ proteins^10–12^. A major impediment in the mammalian DNA repair field has been the random nature of ionizing radiation (IR) or chemical-induced DNA damage, making it impossible to characterize how DNA DSB repair is influenced by chromatin context e.g., regions of coding (euchromatin/gene-rich/H4K16ac-rich) or intergenic noncoding (heterochromatin/gene-poor/H4K16ac-poor) regions^13^. We have circumvented this problem by using two different approaches: (1) using CRISPR/Cas9-based technology to introduce I-SceI cleavage sites and DSB repair cassettes at defined chromosomal sequences to measure DNA DSB repair^14,15^, and; (2) direct induction of a DNA DSB at defined sites in specific phases of cell cycle by using liposomes loaded with CRISPR Single-guide RNA (gRNA)^15^. Using these technological approaches, we report that DSB repair by the HR pathway takes place preferentially at higher frequency where H4K16ac is elevated in gene-rich transcribing regions during the S-phase of the cell cycle.

## Results

### Repair cassette insertion has minimum impact on histone status

**Fig. 1:**
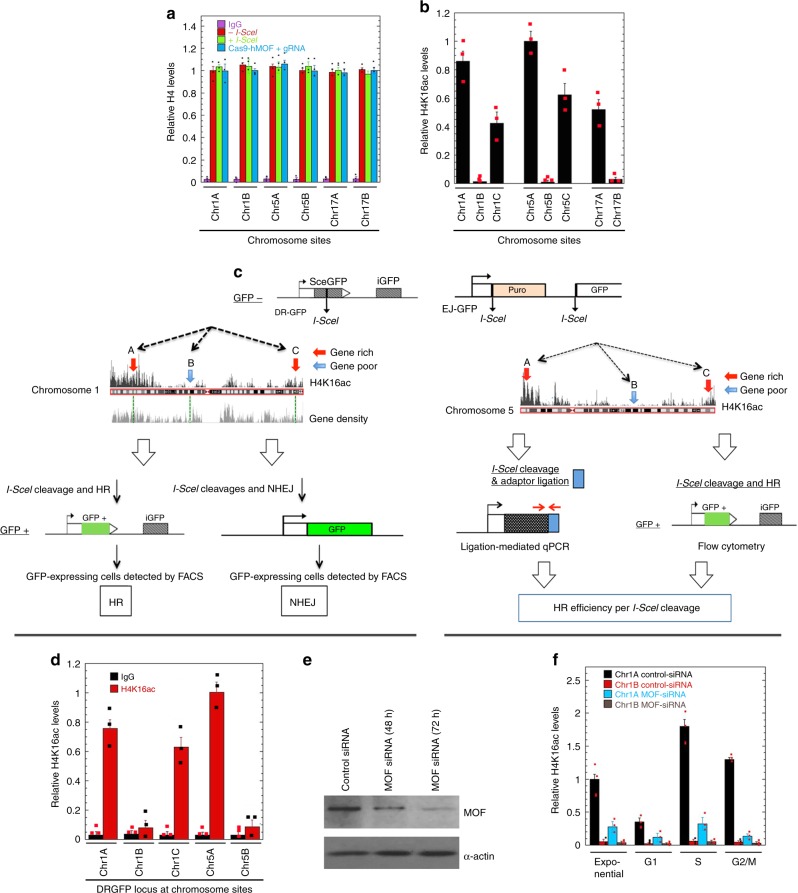
Histone status and repair cassette insertion sites. H4K16ac levels and DNA cleavage efficiency at the targeted human chromosome sites (described in Supplementary Figs. 1 and 3) in H1299, 293, and Hela cells. **a** Histone H4 levels at different sites with and without I-SceI induced DNA cut and with and without expression of dCas9-hMOF with gRNA in H1299 cells. The position of the sites is identified as Chr1A i.e., site A on chromosome 1 as shown in Supplementary Fig. 3. **b** H4K16ac levels at different native chromosome sites in exponentially growing cells were determined by ChIP/PCR and are represented as the relative levels. **c** The DR-GFP (for HR) or EJ5-GFP (for NHEJ) cassettes are inserted at the CRISPR-cleaved sites and a DSB is being induced by the expression of the I-SceI enzyme. GFP-positive cells resulting from HR and or NHEJ are measured by FACS. **d** H4K16ac levels at DR-GFP test genes inserted at different chromosome sites. **e** Western blot showing depletion of MOF with specific siRNA. **f** H4K16ac levels at different stages of the cell cycle

To test whether preexisting H4K16ac levels regulate the frequency of DNA DSB repair by HR, specific sites on three human chromosomes (1, 5, and 17) were chosen for the study (Supplementary Fig. 1)^13^. Based on the relative location of genes and long intergenic regions, we selected three sites on chromosome 1 (Chr1A, Chr1B, and Chr1C); three sites on chromosome 5 (Chr5A, Chr5B, and Chr5C); and two sites on chromosome 17 (Chr17A and Chr17B) (Supplementary Fig. 1) showing similar levels of histone H4 but different levels of H4K16ac (Fig. 1a, b) in order to analyze the impact of chromatin status on DNA DSB repair. To measure HR, a DR-GFP cassette^16^ was inserted in cells at Chr1A, Chr1B, Chr1C, Chr5A, Chr5B, Chr5C, Chr17A, and Chr17B sites (Fig. 1c, Supplementary Table 1). The EJ5-GFP test cassette was inserted into cells at Chr1A and Chr1B sites^17^ to measure NHEJ mediated repair (Fig. 1c, Supplementary Table 1). The insertion sites at Chr1A, Chr1C, Chr5A, Chr5C, and Chr17A are between coding genes, whereas the insertion sites at Chr1B, Chr5B, and Chr17B are within the long noncoding intergenic regions and Chr17B is between KIF2B and TOM1L1 (Supplementary Fig. 2). In parallel, a single I-SceI endonuclease recognition sequence element without reporter cassette was inserted in another set of cell lines at the Chr1A, Chr1B, Chr5A, Chr5B, Chr17A, and Chr17B sites (Supplementary Table 1, Supplementary Fig. 2).

We determined histone H4 levels at the Chr1A, Chr1B, Chr5A, Chr5B, Chr17A, and Chr17B sites before and after DSB induction and did not detect any significant changes in histone H4, even after gRNA tethering of dCas9-hMOF at the sites (Fig. 1a). We subsequently determined H4K16ac levels before and after DR-GFP insertion at the defined regions and found that the relative abundance was unaltered, but observed amongst the sites gene-rich regions generally contained higher H4K16ac levels (Fig. 1d). Overall H4K16ac levels peak in S and G2/M phase cells, while hMOF depletion reduced H4K16ac in all phases of the cell cycle (Fig. 1e, Supplementary Fig. 3).

### Impact of DSB induction on histone status

**Fig. 2:**
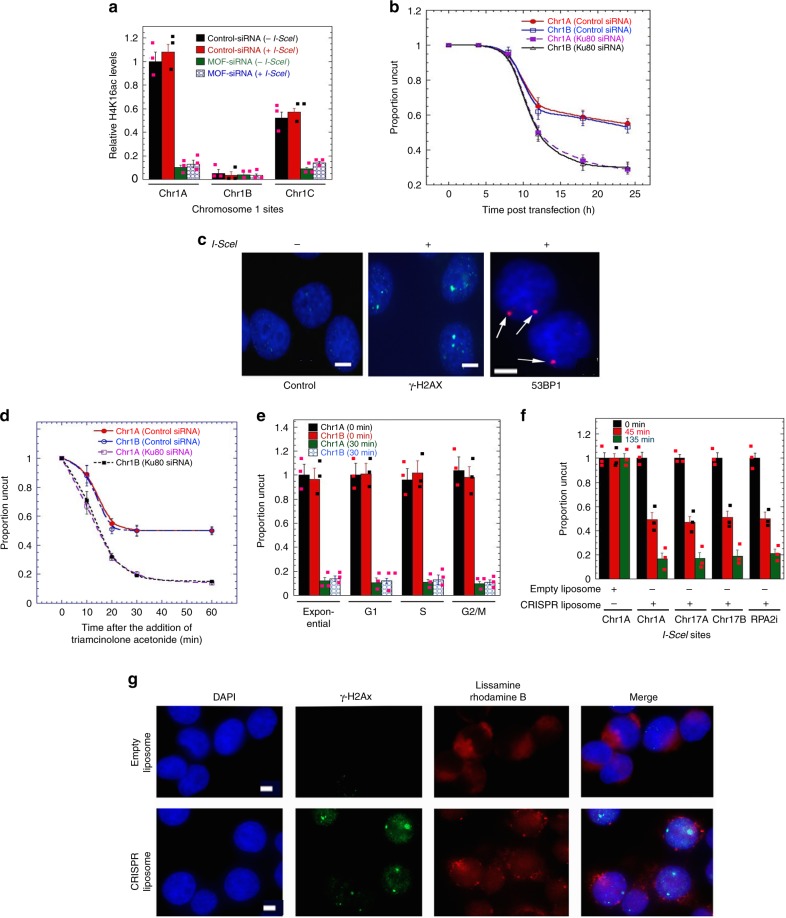
Kinetics of site-specific cleavage. **a** Depletion of MOF with specific siRNA results decrease in H4K16ac levels examined before and after induction of I-SceI induced DSB at different sites (Chr1A, Chr1B, and Chr1C) of chromosome 1 in H1299 cells. **b** PCR-based determination of the uncut DNA at different time points after transfection of the plasmid expressing I-SceI enzyme in cells with and without depletion of KU80. **c** Immunofluorescent detection of γ-H2AX and 53BP1 foci induced by I-SceI DNA cleavage (Scale: 5 μm). **d** Induction of DNA breaks after addition of triamcinolone acetonide to cells with and without knockdown of KU80 with specific siRNA. **e** Comparison of I-SceI cut at different phases of the cell cycle. **f**, **g** Induction and visualization of DSB after cell incubation with CRISPR gRNA liposomes. **f** Ligation-mediated PCR at the indicated times after CRISPR gRNA liposomes addition to measure proportion of uncut DNA. **g** Detection of γ-H2AX foci for site (Chr1A) of chromosome 1 by immunostaining after 45 min of CRISPR gRNA liposome treatment. Nuclei appear as blue (DAPI staining) and liposomes are stained red based on the incorporation of a fluorescent phospholipid lissamine rhodamine B (Scale: 5 μm)

Induction of DSBs did not significantly alter H4K16ac levels in gene-rich regions at the sites examined (Fig. 2a). The efficiency of I-SceI induced DNA DSBs was identical between sites in gene-rich/H4K16ac rich (Chr1A) and gene-poor/H4K16ac poor (Chr1B) (Fig. 2b). DSB levels were increased at both sites after KU80 depletion (Fig. 2b). The DNA cleavage was confirmed by detection of γ-H2AX or 53BP1 foci in cells 15 h post transfection with I-SceI expression vector (Fig. 2c). Alternatively, an I-SceI nuclear translocation system induced DNA breaks within 15 min of drug treatment^18^, with similar cutting efficiencies obtained throughout the cell cycle (Fig. 2d–e). Similar DNA break kinetics were also obtained using a newly developed CRISPR gRNA liposomes to induce the DSB (Fig. 2f–g, Supplementary Fig. 4). All three approaches indicated that, among the selected sites, no significant difference was observed in DNA DSB induction between gene-rich and gene-poor regions (Fig. 2a–g, Supplementary Fig. 4).

### Insertion of cassettes has minimum impact on DNA damage response

**Fig. 3:**
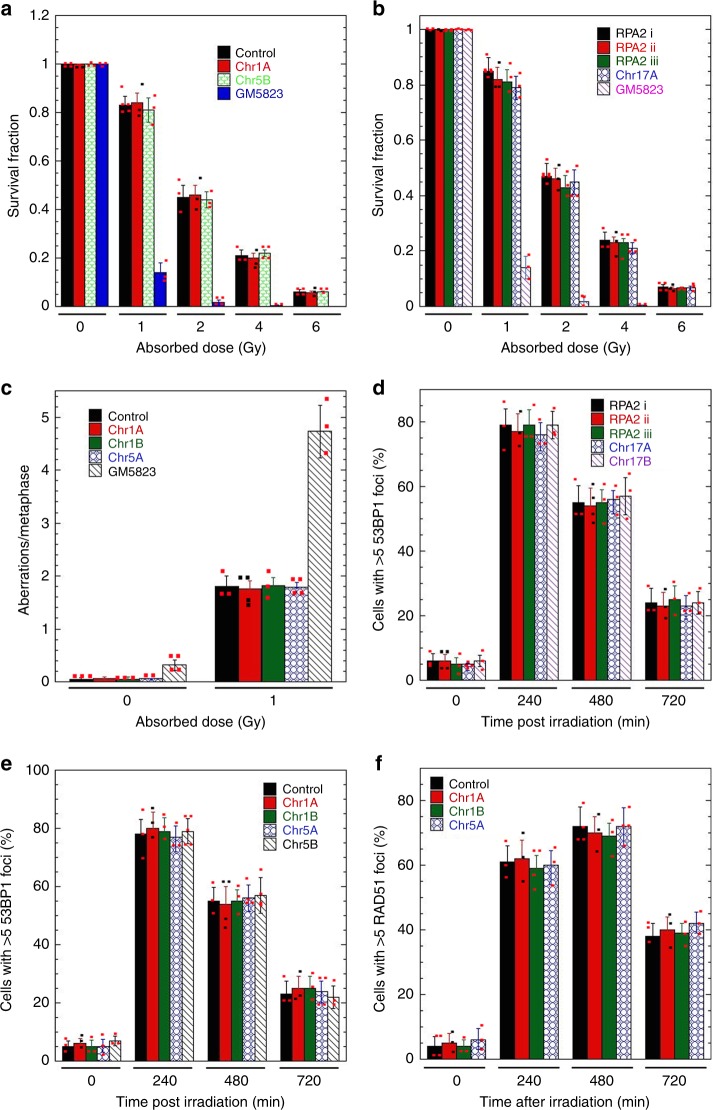
DNA damage response unaltered by insertion of repair cassettes. Cell survival and DNA damage response in cells with inserted I-SceI sequence or DR-GFP or EJ5-GFP cassettes in H1299 cells. **a** Clonogenic cell survival without DR-GFP (control) and with DR-GFP cassettes at different sites (Chr1A and Chr1B) in H1299 and with the positive control as ATM deficient cells (GM5823). **b** Clonogenic cell survival with I-SceI at different sites (RPA2i, RPA2ii, RPA2iii, and Chr17A) in H1299 with the positive control as ATM deficient cells (GM5823). **c** Metaphase aberrations scored at metaphase after 60 min post irradiation in cells with inserted I-SceI sequence. **d** Cells with 53BP1 foci at different time points post irradiation with 5 Gy in cells inserted with I-SceI sequence at different sites (RPA2i, RPA2ii, RPA2iii, Chr17A, and Chr17B). **e** 53BP1 foci analysis at different time points post irradiation with 5 Gy in cells without (control) and with inserted with DR-GFP cassette at different sites (Chr1A, Chr1B, Chr5A, and Chr5B). **f** Cells with RAD51 foci at different time points post irradiation with 5 Gy in cells without (control) and with inserted with DR-GFP cassette at different sites (Chr1A, Chr1B, and Chr5A)

Relative frequency of DNA DSB repair by NHEJ and HR functions were measured in cells by repair-dependent reconstitution of disrupted GFP repair cassettes after DSB induction by ectopic I-SceI endonuclease expression^19,20^. First, we determined whether the insertion of I-SceI sequences, DR-GFP or EJ5-GFP cassettes (Fig. 1c,d, Supplementary Fig. 2) impacts the global DNA damage response. Clonogenic survival, repairosome formation, and residual chromosome aberrations after IR exposure were all unaltered in the different cell lines (Fig. 3). This suggests that introduction of I-SceI elements or DNA repair reporter cassettes did not impact global DNA damage sensing, response, and repair.

### H4K16ac levels impact DSB repair by HR in gene-rich regions

Since there was no difference in DNA DSB induction between different targeted sites, we next examined whether the repair pathway at the different sites was influenced by the context of a high or low H4K16ac density. We first compared DSB repair by NHEJ at different insertion sites with and without ligase IV depletion and found DSB repair efficiency to be similar irrespective of the H4K16ac status (Supplementary Fig. 5).

**Fig. 4:**
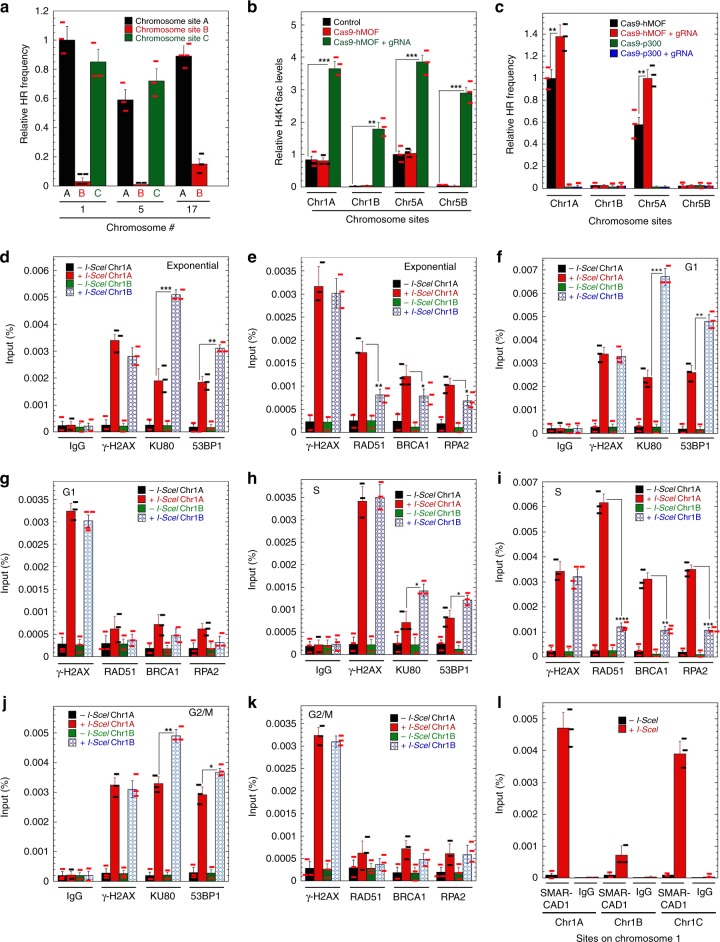
Measurement of HR and repairosome protein recruitment to different genomic sites. **a** DR-GFP cassettes were inserted at different positions on the chromosome and the histograms present HR frequency at the sites. Chromosome sites designated as A, B, and C are Chr1A, Chr1B, Chr1C, Chr5A, Chr5B, Chr5C, Chr17A, and Chr17B. **b** Tagging of dCas-9-hMOF and gRNA at different sites increases the H4K16ac levels at all the sites. **c** Comparison of HR frequency at different sites among the cells with and without dCas-9-hMOF + gRNA. (**d**–**k** Detection of repair proteins by ChIP at different I-SceI sites before and after DSB induction. Cell synchronization, cell cycle analysis, I-SceI-induced DSB, and ChIP analysis were done according to the described procedure^19,38,44^. **d** Exponential cells for NHEJ proteins; **e** exponential cells for HR proteins; **f** G1 cells for NHEJ proteins; **g** G1 cells for HR proteins; **h** S cells for NHEJ proteins; **i** S cells for HR proteins; **j** G2/M cells for NHEJ proteins; **k** G2/M cells for HR proteins; **l** exponential cells for SMARCAD1 protein. (*P < 0.05; **P < 0.01 and ***P < 0.001, determined by the chi-square test)

We next examined whether chromatin status impacts HR-mediated DSB repair (DR-GFP inserted sites) (Supplementary Table 1, Supplementary Fig. 2) and found higher HR repair in gene-rich chromatin regions associated with higher H4K16ac levels as compared to the three different gene-poor chromosomal regions (Fig. 4a, Supplementary Fig. 6). Since there was a good correlation between H4K16ac and HR levels, we tested whether increasing local H4K16ac levels in gene-poor regions would increase HR repair frequency. A nuclease-dead dCas9-hMOF fusion protein (dCas9-hMOF) was ectopically expressed and tethered immediately upstream of the DR-GFP cassettes by coexpressing a guide RNA (gRNA) specific for each locus (Supplementary Fig. 7). Measurement by ChIP-qPCR of dCas9-hMOF tethering at the Chr1A, Chr1B, Chr5A, and Chr5B sites indicated successful localization, as judged by a significant increase in local H4K16ac levels (Fig. 4b, Supplementary Fig. 8), without affecting nucleosome occupancy/total H4 levels (Fig. 1a). The local H4K16ac level was not increased significantly by another acetyltransferase construct, dCas9-p300(Core), that does not affect H4K16ac status (Supplementary Fig. 8). The artificially increased local levels of H4K16ac (Fig. 4b) did not affect DNA DSB induction, as measured by LM-PCR analysis of I-SceI digestion efficiency (Supplementary Fig. 9). The HR efficiency at the Chr1A and Chr5A loci was increased significantly by dCas9-hMOF with gRNA, but no increase was detected at the Chr1B and Chr5B sites, indicating that artificially increased H4K16ac impacts HR efficiency only in euchromatic regions (Fig. 4c). Tethering of dCas9-p300 (Core) to DSB sites did not influence HR efficiency at any site (Fig. 4c).

The differences in HR repair frequency at gene-rich and gene-poor sites could be due to altered recruitment of HR-related factors. While there is no significant difference detected in phosphorylated H2AX (γ-H2AX) at the DSB sites, higher KU80 and 53BP1 signals are observed at Chr1B, whereas RAD51, BRCA1, and RPA2 levels are higher at Chr1A in exponentially growing cells (Fig. 4d–e). A cell cycle-regulated circuit, underpinned by RIF1 and BRCA1, governs DSB repair pathway choice to ensure that NHEJ dominates in G1 and HR is favored from S-phase onward^21^. We also found that in G1-arrested cells, KU80 and 53BP1 are increased at the Chr1B site, (Fig. 4f–g), whereas RAD51, BRCA1, and RPA2 levels are higher at Chr1A site in S-phase cells (Fig. 4h–i). Recruitment of repair proteins at Chr1A and Chr1B DSB sites in G2/M cells is similar to what is seen in G1 cells (Fig. 4j–k).

What favors the recruitment of HR proteins to DSB sites in high H4K16ac regions is not clear. SMARCAD1 protein is recruited to newly synthesized DNA and facilitates histone deacetylation, histone H3K9 trimethylation (H3K9me3), and efficient HP1 recruitment through a mechanism coupled to ATP hydrolysis^22^. SMARCAD1 is also recruited to DNA DSBs during DNA resection through an ATM-dependent process and has been suggested to assist nucleosome displacement during the resection process leading to HR^19,23^. Our analysis of SMARCAD1 recruitment to DSBs indicates enrichment is significantly higher at DSB sites located in gene-rich regions (Chr1A and Chr1C), compared to the Chr1B gene-poor region (Fig. 4l). A similar significant increase of SMARCAD1 levels is also seen at Chr17A after DSB induction with CRISPR liposomes at 120 min of treatment, compared to Chr17B (Supplementary Fig. 10).

### RNA pol II and CSB loading at DSB sites is higher in gene-rich regions

**Fig. 5:**
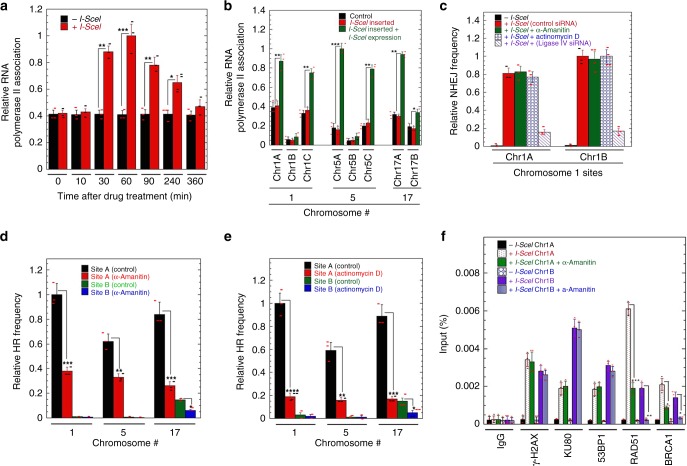
Impact of a DSB on RNA polymerase II loading at sites with different H4K16ac levels. **a** Kinetics of RNA polymerase II loading and dissociation after DSB induction. **b** RNA polymerase II levels before and after induction of DSB at the different sites on chromosomes. **c** α-amanitin or actinomycin D treatment does not alter NHEJ frequency at sites with either high or low levels of H4K16ac. **d** α-amanitin treatment effects on the relative HR frequency. **e** Actinomycin D treatment effects on the relative HR frequency. **f** Effect of α-amanitin on recruitment of proteins at different I-SceI sites before and after induction of DSB. (*P < 0.05; **P < 0.01; ***P < 0.001 and ****P < 0.0001, determined by the chi-square test)

Transcription is a critical factor in deciding which pathway is used for DSB repair^24^ as RNA polymerase II (RNAPII) recognizes DNA damage^25^. We observed that local RNAPII levels increase at the DSB site after break induction in gene-rich regions (Fig. 5a–b). While treatment of cells with drugs-blocking transcription (α-amanitin or actinomycin D) has little effect on DSB repair by NHEJ (Fig. 5c), repair by HR repair is significantly decreased at the Chr1A DSB site with no effect on Chr1B (Fig. 5d–e). Consistent with these results, α-amanitin treatment has no impact on KU80 or 53BP1 recruitment at DSB sites, whereas RAD51 and BRCA1 recruitment is significantly reduced at the Chr1A DSB site (Fig. 5f).

**Fig. 6:**
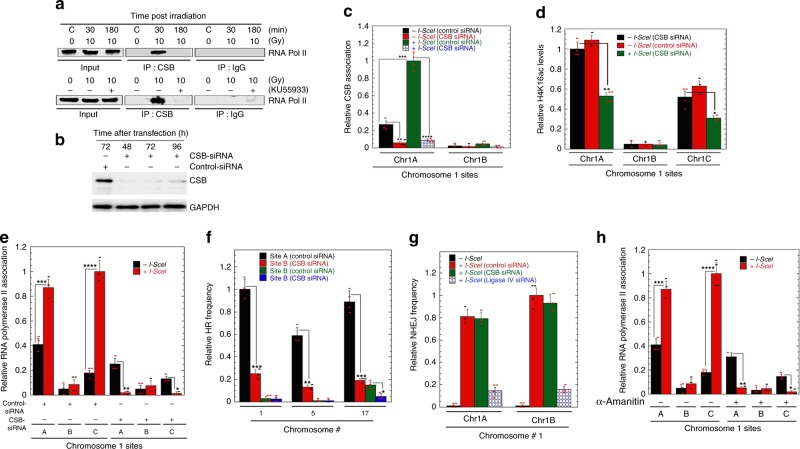
Impact of RNA polymerase II and CSB on DSB repair. **a** Interaction between RNA polymerase II and CSB after cells being irradiated with 10 Gy in presence and absence of ATM inhibitor (KU55933) in H1299 cells. **b** Western blot showing CSB depletion at different times after transfection with specific siRNA. **c** CSB levels before and after induction of DSB at the different sites on chromosome 1. **d** Impact of CSB depletion on H4K16ac levels before and after DSB induction. **e** Impact of CSB depletion on RNA polymerase II loading at the DSB sites (A = Chr1A, B = Chr1B, and C = Chr1C). **f** Effect of CSB depletion on HR frequency at the different chromosomal locations (Site A = Chr1A, Chr5A, and Chr17A; Site B = Chr1B, Chr5B, and Chr17B). **g** Comparison of the relative frequency of DSB repair by NHEJ at the different sites on chromosome number 1 (Chr1A and Chr1B) with and without depletion of ligase IV. **h** Impact of α-Amanitin treatment on the recruitment of RNA polymerase II at the different sites DNA of chromosome 1 (A = Chr1A, B = Chr1B, and C = Chr1C) with and without induction of DSB. (*P < 0.05; **P < 0.01; ***P < 0.001 and ****P < 0.0001, determined by the chi-square test)

How HR-mediated DSB repair is influenced by transcription could relate to the CSB remodeler, which is an essential factor in transcription-coupled nucleotide excision repair (NER)^24,26–28^. We tested whether CSB interacts with RNAPII post DNA DSB induction and observed association in an ATM-dependent manner (Fig. 6a). Moreover, CSB levels increased after DSB induction only at DSB sites in the gene-rich regions (Fig. 6b–c). Interestingly, depletion of CSB (Fig. 6b) reduced H4K16ac levels after DSB induction (Fig. 6d), led to failure of RNAPII accumulation at the DSB sites (Fig. 6e), and decreased HR (Fig. 6f, Supplementary Fig. 11), without affecting repair by NHEJ (Fig. 6g). Thus, when cells are treated with α-amanitin, stalled RNAPII is reduced at DSBs (Fig. 6h), and there is reduced recruitment of HR-related factors (Fig. 5f) with failure of HR-mediated repair (Fig. 6e). These observations support the argument that RNAPII-mediated transcription is critical for DSB repair in gene-rich/H4K16ac rich regions. This is in agreement with a previous report that HR is linked to the H3K36me3 chromatin mark deposited on transcribed genes in an RNAPII-dependent manner^29^. Interestingly, hMOF is a part of the MSL complex, which contains a MSL3 subunit with a chromodomain specific for H3K36me3^2,30^.

### RNA pol II and CSB load on *RPA2* gene DSB sites during repair

**Fig. 7:**
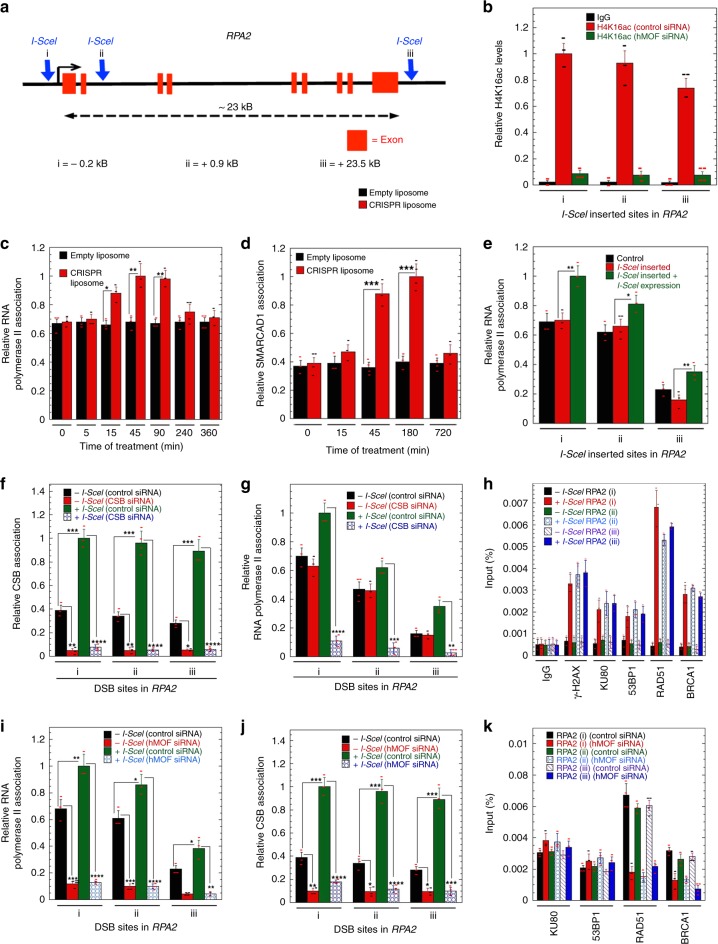
Loading of RNA polymerase II and CSB on *RPA2* during repair. **a** I-SceI sites were introduced by CRISPR at the indicated positions in the human *RPA2* gene (H1299 cells) using single-stranded oligonucleotides carrying the 18nt I-SceI site flanked with 45–90 bp homologous sequence. **b** H4K16ac levels at the different I-SceI sites of RPA2. **c** RNA polymerase II loading at site (**i**) of *RPA2* after DSB induction. **d** Kinetics of SMARCAD1 association at I-SceI site of *RPA2* (**i**) after DSB induction. **e** RNA polymerase II loading at three different I-SceI sites on *RPA2* after induction of DSB. **f** CSB loading at the three different sites of *RPA2* before and after induction of DSB. **g** Depletion of CSB impacts the loading of RNA polymerase II after induction of DSB at different sites of RPA2. **h** Recruitment of repairosome proteins at the DSB sites of *RPA2* gene before and after induction of DSB. **i** MOF depletion by specific siRNA impacts the RNA polymerase II loading before and after induction of DSB at different sites of RPA2 gene. **j** MOF depletion by specific siRNA impacts the CSB loading before and after induction of DSB at different sites of *RPA2* gene. **k** Association of repairosome protein recruitment to DSB sites after I-SceI expression in the *RPA2* gene in cells depleted for MOF. (*P < 0.05; **P < 0.01; ***P < 0.001 and ****P < 0.0001, determined by the chi-square test)

We consistently observed that HR frequency was higher in gene-rich/transcribed regions (euchromatin) as compared to nontranscribing regions. We tested whether different regions of a transcribing gene might be subject to preferential DSB repair. We selected the *RPA2* gene for analysis and introduced I-SceI element (18 bp) at three different locations (Fig. 7a). Measured H4K16ac levels at the I-SceI sites were highest (i) in the promoter region and lowest (iii) at the end of *RPA2* gene (Fig. 7b). All three DNA sites had similar I-SceI cleavage efficiencies with similar RPA2 protein levels (Supplementary Fig. 12). An increase in RNAPll and SMARCAD1 enrichment peaked around 45 min after DSB induction (Fig. 7c, d) with maximum levels and increased RNAPII association observed at the promoter DSB site (i) (Fig. 7e). CSB interacts with RNAPII and we found that CSB levels were also higher at the promoter site, increasing upon DSB induction (Fig. 7f). To determine whether accumulation of RNAPII is dependent on CSB, we depleted CSB and found decreased RNAPII/DNA signals, with no increase after DSB induction (Fig. 7g).

Consistent with the RNAPII results, SMARCAD1 enrichment after DSB induction was also maximal at the promoter region I-SceI site **(**Supplementary Fig. 13). When we examined repair proteins levels at the three different *RPA2* gene sites, all had increased enrichment of HR-related factors after DSB induction, supporting the concept that the entire transcribed region is preferentially repaired by HR (Fig. 7h). Since H4K16ac status is coupled with transcriptional activation, we determined whether depletion of hMOF impacts H4K16ac levels in the *RPA2* gene. As expected, MOF-specific knockdown reduced H4K16ac signal at all three sites along the *RPA2* gene (Fig. 7b). Consistent with the reduced H4K16ac levels, RNAPII before and after DSB induction was also significantly reduced (Fig. 7i), as was CSB binding. (Fig. 7j). Reduction of H4K16ac had no effect on NHEJ protein recruitment, while recruitment of HR factors RAD51 and BRCA1 was significantly reduced after DSB induction (Fig. 7k). These results further indicate that transcriptionally active regions with higher H4K16ac levels preferentially repair DNA DSBs using the HR pathway, preserving the integrity of the most important sequences in the genome, the coding ones.

## Discussion

The chromatin flanking a DNA DSB undergoes extensive post-translational modification, such as phosphorylation of H2AX^31^ at the initial stage and more other protein modifications during DSB repair^32,33^. In addition, histones are removed from around DSBs to enable DNA repair and, upon completion of repair, are restored to reestablish the initial chromatin structure^32,34^. The H4K16ac modification has been reported to modulate both higher order chromatin structure and functional interactions between a nonhistone protein and the chromatin fiber^7^. The histone H4K16ac mark is established by the MOF protein, which has also been shown to interact with a range of proteins that extend its potential significance well beyond transcription^35^. For example, MOF is an upstream regulator of the ATM (ataxia–telangiectasia mutated) protein, and MOF loss impacts ATM function that can result in an ataxia–telangiectasia (AT)-like neurological phenotype^36,37^. Conversely, ATM can also regulate MOF function through post-translational modifications^38^.

A major impediment in the mammalian DNA repair field to answering how DSB repair takes place within the context of chromatin status is the nonspecificity of DNA damage induced by agents, making it difficult to characterize how specific differences in chromatin environment impact recruitment of DNA lesion signaling/repair factors. We have circumvented this problem by using site-specific DSB induction in combination with a high-density genome wide map identifying H4K16ac-rich or poor chromosomal sites^13^. Utilizing this data, we directly tested the hypothesis that local chromosomal H4K16ac levels impact the recruitment of proteins involved in either major DSB repair pathway, as well as determined the frequency of HR in gene-rich and gene-poor regions. Thus we were able to show that genomic regions with elevated preexisting H4K16ac histone levels, which are linked with transcription, are associated with preferential recruitment of HR-related DSB repair proteins and an increased frequency of DSB repair by HR. Consistent with the role of H4K16ac in preferential repair in transcribed regions, we report that H4K16ac rich regions have higher levels of RNAPII and CSB whose inhibition/depletion reduces DSB repair by HR. MOF is the major enzyme acetylating histone H4 at K16, and its role in transcription and the DNA damage response is conserved among insects and mammals^3,35,39,40^. Active transcription and Rad52 recognition of associated R-loops selects a critical portion of DSBs for HR-mediated repair^41^. These sites may be further differentiated by a high density of preexisting H4K16ac marks since this chromatin modification also has a profound effect on the DNA structure^7^, as well as transcriptional functions^6^. While HR is thought to be the most efficient means for maintaining transcribed gene fidelity during DNA repair, a multi-invasion-induced rearrangement occurring during HR was recently identified which uniquely amplified the initial DNA damage and possibly increased genome rearrangements^42^. Long-read sequencing and improved mapping of repeats should enable better appreciation of the significance of HR-related recombination in generating genomic rearrangements. In summary, by using a combination of technologies and producing site-specific DSBs within a defined chromatin context, we have addressed a very important question that opens a new area of research into the DNA damage response and its interaction with the preexisting chromatin status.

## Methods

### Cell lines

H1299 (human nonsmall cell lung carcinoma cell line), HEK293 (Human embryonic kidney 293 cells), and U2OS (Human bone osteosarcoma epithelial cells) and HeLa cell lines (ATCC) were grown in DMEM (Sigma) and 10% fetal calf serum (Sigma) supplemented with 1% penicillin/streptomycin.

### Clonogenic assay

Cells were irradiated with graded doses of X-rays as described previously^19^ and incubated for 10–14 days. Colonies were stained with 0.5% crystal violet (Gibco) in 20% methanol with 1% formaldehyde and counted. Each individual group was processed in triplicate and normalized to untreated controls. The survival graphs show combined data from at least three to four independent experiments, and bars show standard error.

### Antibodies

Antibodies for histone H4 (Millipore, 05858), H4K16ac (Millipore, 07329) SMARCAD1 (Santa Cruz Biotech, sc-162233, Novus Bio NB100–79835), γ-H2AX (Millipore, JBW301), Ku80 (Cell Signaling, 2180P), RAD51 (Abcam, ab213), BRCA1 (Santa CruzBiotech,sc-642), 53BP1 (Santa Cruz Biotech, sc-22760), CSB (Santa Cruz, 25371, 166042), GAPDH (Protein Tech, HRP-6004), were used for western blotting, immunoprecipitation and immunofluorescence. Antibodies used for the chromatin immunoprecipitation (ChIP) assay were histone H4 (Millipore, 05858), H4K16ac (Millipore, 07329), SMARCAD1 (Abnova, H00056916-BO1P), γ-H2AX (Millipore, JBW301), 53BP1 (Santa Cruz Biotech, sc-22760), BRCA1 (Abcam), Ku80 (Thermo Fisher,MA5–12933), RAD51(Abcam,ab176458), RAD51 (Abcam, ab176458), and RNA polymerase II (Abcam, ab817).

### Generation of CRISPR/Cas9-based cell line

gRNA against the sites described in Supplementary Figs. 1 and2, was designed by screening the target sequence with the online tool http://www.broadinstitute.org/rnai/public/analysis-tools/sgrna-design^15^. One high-score gRNA target sequence was detected and then targeted on the sense strand sequence (as shown in Supplementary Fig. 2). The gRNA module was generated by overlapping PCR protocol with minor modifications, and was subsequently cloned into the same pLX-sgRNA vector. Transfection of humanized Cas9 that contained lentiviral pCW-Cas9 and customized pLX-sgRNA plasmids into H1299 cells^15^, and cells were selected by Zeocin (505 µg/mL) and blusticidin (5 µg/mL).

### gRNA generation by in vitro transcription

CRISPR gRNA was designed based on the sequence from^43^ and template oligonucleotides with reverse complement sequence including T7 promoter were ordered from Eurofins Genomics (Louisville, KY). Template oligonucleotides were transcribed into gRNA using the MEGAscript™ T7 Transcription Kit (Invitrogen, Carlsbad, CA) according to manufacturer’s protocol. gRNA was purified using Oligo Clean & Concentrator^TM^ (Zymo Research, Irvine, CA) according to manufacturer’s procedures. For concentration determination, produced gRNA was diluted 1:20 in nuclease-free water and measured using Take3 plates and Synergy H4 Hybrid Reader (Biotek, Winooski, VT).

gRNA sequences are shown in Table 1.

**Table 1:** gRNA sequences

Locus	gRNA sequence
Chr1A	GCCCCCAAUAACAAAAUCGAguuuuagagcuagaaauagcaaguuaaaauaaggcuaguccguuaucaacuugaaaaaguggcaccgagucggugcuuuu
RPA2i	AAUUUUCCACCUCCUGUUUguuuuagagcuagaaauagcaaguuaaaauaaggcuaguccguuaucaacuugaaaaaguggcaccgagucggugcuuuu
Chr17A	GGAGGAUGGCCCAAUGUCGCguuuuagagcuagaaauagcaaguuaaaauaaggcuaguccguuaucaacuugaaaaaguggcaccgagucggugcuuuu
Chr17B	GAGCUGAUCUCUCCUACCUUguuuuagagcuagaaauagcaaguuaaaauaaggcuaguccguuaucaacuugaaaaaguggcaccgagucggugcuuuu

### CRISPR gRNA liposomes design and characterization

Liposomes were prepared by lipid hydration-extrusion method. 3.62 mg soybean phosphatidylcholine (Lipoid S100, Lipoid, Germany), and 0.88 mg 1,2-dioleoyl-3-trimethylammonium-propane (chloride salt) (Avanti, Alabama, USA) were dissolved in 3 mL ethanol. To fluorescently label liposome, fluorescent phospholipid lissamine rhodamine B 1,2-dihexadecanoyl-sn-glycero-3-phosphoethanolamine, triethylammonium salt (rhodamine-DHPE, Invitrogen) was incorporated in the membrane at 2% of the total lipid to all liposome formulations.

Thin film was formed by evaporating the solvent for 30 min at 41 °C/ 150 rpm using rotary evaporator (Rotavapor, Buchi, Switzerland). The thin film was rehydrated with 0.5 mL PBS pH 7.2, followed by the sonication of the liposomes. Liposomes were sonicated intermittently for 15 min until the desired size was achieved using Branson 1510 (Branson, Danbury, CT).

CRISPR gRNA liposomes were prepared by adding 1 μg GeneArt^TM^ Platinum^TM^ Cas9 nuclease (Thermo Scientific, Vilnius, Lithuania) to 400 ng gRNA in 50 μL serum free MEM medium. After incubation at room temperature for 5 min, 2 μL were added to liposomes were added to the Cas9-gRNA complex and incubated for 5 min prior to transfection. CRISPR gRNA liposomes’ size (164.4 nm, polydispersity index 0.15) and zeta potential (23.93 ± 1.48 mV) were assessed in triplicates by dynamic light scattering using Zetasizer instrument (Malvern, Worcestershire, UK).

### Western blotting and Immunoprecipitation

Cell lysates preparation and western blotting were performed as described previously^38^. The cell extract immunoprecipitation procedure is the same as described previously^36^. Full blots corresponding to Figs. 1e and 6 are shown in Supplementary Figs. 14 and 15.

### ChIP assay

Chromatin immunoprecipitation assays were carried out using the previously described standard procedures^19,38,44^. Cells were synchronized as described previously^44^, transfected with I-SceI endonuclease expression vector or treated with the drug or liposomes as described^19,38,44^. After protein–DNA crosslinking, the chromatin was centrifuged, the supernatant collected and diluted (1:10) with ChIP dilution buffer as described previously^19^. Diluted chromatin was incubated with specific antibody and Magna ChIP Protein A/Gbeads (Millipore) overnight at 4 ^°^C, subsequent steps were performed to reverse the crosslinking and the DNA was purified using QIAquick Spin columns (Qiagen). qPCR was carried out with specific sets of primers at the proper melting temperatures. Each experiment was repeated 3–4 times with consistent results. The signal to input ratio was low but significantly higher in comparison to IgG control values. Similar low ratios have been reported by other investigators^19,45^ and, in this case, likely reflect technical limits on detection of a protein bound to a single DSB site (the single I-SceI site) in the entire genome of the cell. RPA2 primers around I-SceI sites are shown in Table 2.

**Table 2:** RPA2 primers around I-SceI sites

Primer	Sequence
RPA2i (F)	GCAAGAGGGCGTAGGTTTCT
RPA2i (R)	AGGTTCGCGCCAAACTATCA
RPA2ii (F)	TCCTGTCCTGAAGTCCCTGA
RPA2ii (R)	GGGCGTTCACACAAGACTGA
RPA2iii (F)	GAAAACGTTATTTCCCCTGGGT
RPA1iii (R)	AGCCAATCAAGTTGGCCAAAAA
Chr1A (F)	CTGAGTGTGGGAAGCTCTGG
Chr1A (R)	GGAAGCAGTCTTCTCCGCTT
Chr1B (F)	TACCCTCAGCTCCTGTACCC
Chr1B (R)	GAGAGAAGCAAAGTTGCCAGCC
Chr1C (F)	CCTGTTGTCCGGAAACTGCTC
Chr1C (R)	CCTCGACTTTCCTGGGTGATTG
Chr5A (F)	CACATTCACCAGCTCTCATGC
Chr5A (R)	GCTCCATCAAGGCCATCTTA
Chr5B (F)	CAGTTCCTTATGTGTATGTCACCTG
Chr5B (R)	AAAGCAGATTTCATGGAGAATGTAA
Chr5C (F)	GTGAGAAAATGCTAGAGCTAGTGG
Chr5C (R)	GATGCAGACTCTGTCCTTGGAG
Chr17A (F)	CTGCTCTCCAACAGGCCAG
Chr17A (R)	CCCACCCCTCTTCTTTCCAG
Chr17B (F)	AGAAGCAGTGACACGATGGA
Chr17B (R)	GTGTCAAAGCAACTTGGGCC
DRGFP (F)	AAGATCCGCCACAACATCGA
DRGFP (R)	GTCCATGCCGAGAGTGATCC

### DSB repair by NHEJ and HR assay

The DSB repair assay by NHEJ or HR was performed by the procedure described previously^19^. To perform the NHEJ assay, commercially available EJ5 GFP-Puro plasmid DNA was integrated at the different sites of chromosome 1. The HR assay was performed in H1299 cells with a stably integrated DR-GFP cassette at different sites as described previously^19,46,47^. The percentage of GFP-positive cells after I-SceI DSB induction was measured by flow cytometry and used to define NHEJ or HR repair.

The relative values of HR were determined by counting cells positive for GFP at the site of chromosome 1 A (Chr1A), which gave the maximum percentage of positive cells and this is considered as 1. In the HR assay, cells positive for GFP are counted at other sites (Chr1B, Chr1C, Chr5, and Chr17) and plotted relative to the value obtained for Chr1A. In the NHEJ assay, the maximum cells positive for GFP are observed at chromosome 1 site B (Chr1B) and relative values are plotted.

### Chromosome aberrations

Chromosomal aberrations at metaphase were examined as previously described^46^. Fifty metaphases were scored for each experiment and each experiment was repeated three to four times.

### Statistics

Data were expressed as mean ± SD from three to four different experiments, and were analyzed by two-tailed unpaired Student’s *t* test. Statistical significance was assessed at *p < 0.05, **p < 0.01, ***p < 0.01 and ****p < 0.0001.

### Reporting summary

Further information on research design is available in the Nature Research Reporting Summary linked to this article.

## Supplementary information

Supplementary Information

Reporting Summary

## Data Availability

The data that support the findings of this study are available from the corresponding author upon reasonable request.

## References

[1] Smith CM (2003). Mass spectrometric quantification of acetylation at specific lysines within the amino-terminal tail of histone H4. Anal. Biochem..

[2] Sharma GG (2010). MOF and histone H4 acetylation at lysine 16 are critical for DNA damage response and double-strand break repair. Mol. Cell. Biol..

[3] Taipale M (2005). hMOF histone acetyltransferase is required for histone H4 lysine 16 acetylation in mammalian cells. Mol. Cell. Biol..

[4] Hilfiker A, Hilfiker-Kleiner D, Pannuti A, Lucchesi JC (1997). mof, a putative acetyl transferase gene related to the Tip60 and MOZ human genes and to the SAS genes of yeast, is required for dosage compensation in Drosophila. EMBO J..

[5] Suka N, Luo K, Grunstein M (2002). Sir2p and Sas2p opposingly regulate acetylation of yeast histone H4 lysine16 and spreading of heterochromatin. Nat. Genet..

[6] Akhtar A, Becker PB (2000). Activation of transcription through histone H4 acetylation by MOF, an acetyltransferase essential for dosage compensation in *Drosophila*. Mol. Cell..

[7] Shogren-Knaak M (2006). Histone H4-K16 acetylation controls chromatin structure and protein interactions. Science.

[8] Aymard F (2014). Transcriptionally active chromatin recruits homologous recombination at DNA double-strand breaks. Nat. Struct. Mol. Biol..

[9] Clouaire Thomas, Rocher Vincent, Lashgari Anahita, Arnould Coline, Aguirrebengoa Marion, Biernacka Anna, Skrzypczak Magdalena, Aymard François, Fongang Bernard, Dojer Norbert, Iacovoni Jason S., Rowicka Maga, Ginalski Krzysztof, Côté Jacques, Legube Gaëlle (2018). Comprehensive Mapping of Histone Modifications at DNA Double-Strand Breaks Deciphers Repair Pathway Chromatin Signatures. Molecular Cell.

[10] Chakraborty A (2016). Classical non-homologous end-joining pathway utilizes nascent RNA for error-free double-strand break repair of transcribed genes. Nat. Commun..

[11] Gunn A, Bennardo N, Cheng A, Stark JM (2011). Correct end use during end joining of multiple chromosomal double strand breaks is influenced by repair protein RAD50, DNA-dependent protein kinase DNA-PKcs, and transcription context. J. Biol. Chem..

[12] McDevitt S (2018). How RNA transcripts coordinate DNA recombination and repair. Nat. Commun..

[13] Horikoshi N (2013). Genome-wide distribution of histone H4 lysine 16 acetylation sites and their relationship to gene expression. Genome Integr..

[14] Jacquet K (2016). The TIP60 Complex Regulates Bivalent Chromatin Recognition by 53BP1 through Direct H4K20me Binding and H2AK15 Acetylation. Mol. Cell..

[15] Wang T, Wei JJ, Sabatini DM, Lander ES (2014). Genetic screens in human cells using the CRISPR-Cas9 system. Science.

[16] Nakanishi K, Cavallo F, Brunet E, Jasin M (2011). Homologous recombination assay for interstrand cross-link repair. Methods Mol. Biol..

[17] Gunn A, Stark JM (2012). I-SceI-based assays to examine distinct repair outcomes of mammalian chromosomal double strand breaks. Methods Mol. Biol..

[18] Soutoglou E (2007). Positional stability of single double-strand breaks in mammalian cells. Nat. Cell Biol..

[19] Chakraborty S (2018). SMARCAD1 phosphorylation and ubiquitination are required for resection during DNA double-strand break repair. Iscience.

[20] Mujoo K (2017). Differentiation of human induced pluripotent or embryonic stem cells decreases the dna damage repair by homologous recombination. Stem Cell Rep..

[21] Escribano-Diaz C (2013). A cell cycle-dependent regulatory circuit composed of 53BP1-RIF1 and BRCA1-CtIP controls DNA repair pathway choice. Mol. cell..

[22] Rowbotham SP (2011). Maintenance of silent chromatin through replication requires SWI/SNF-like chromatin remodeler SMARCAD1. Mol. Cell..

[23] Chen X (2012). The Fun30 nucleosome remodeller promotes resection of DNA double-strand break ends. Nature.

[24] Marnef A, Cohen S, Legube G (2017). Transcription-Coupled DNA Double-Strand Break Repair: Active Genes Need Special Care. J. Mol. Biol..

[25] Lindsey-Boltz LA, Sancar A (2007). RNA polymerase: the most specific damage recognition protein in cellular responses to DNA damage?. Proc. Natl Acad. Sci. USA..

[26] Selby CP, Sancar A (1997). Human transcription-repair coupling factor CSB/ERCC6 is a DNA-stimulated ATPase but is not a helicase and does not disrupt the ternary transcription complex of stalled RNA polymerase II. J. Biol. Chem..

[27] Scheibye-Knudsen M (2016). Cockayne syndrome group A and B proteins converge on transcription-linked resolution of non-B DNA. Proc. Natl Acad. Sci. USA..

[28] Boetefuer EL, Lake RJ, Fan HY (2018). Mechanistic insights into the regulation of transcription and transcription-coupled DNA repair by Cockayne syndrome protein B. Nucleic Acids Res..

[29] Pfister SX (2014). SETD2-dependent histone H3K36 trimethylation is required for homologous recombination repair and genome stability. Cell Rep..

[30] Kadlec J (2011). Structural basis for MOF and MSL3 recruitment into the dosage compensation complex by MSL1. Nat. Struct. Mol. Biol..

[31] Iacovoni JS (2010). High-resolution profiling of gammaH2AX around DNA double strand breaks in the mammalian genome. EMBO J..

[32] Berkovich E, Monnat RJ, Kastan MB (2007). Roles of ATM and NBS1 in chromatin structure modulation and DNA double-strand break repair. Nat. Cell Biol..

[33] Huang TH, Shen ZJ, Sleckman BP, Tyler JK (2018). T.he histone chaperone ASF1 regulates the activation of ATM and DNA-PKcs in response to DNA double-strand breaks. Cell Cycle.

[34] Chen CC (2008). Acetylated lysine 56 on histone H3 drives chromatin assembly after repair and signals for the completion of repair. Cell.

[35] Mujoo Kalpana, Hunt Clayton R., Horikoshi Nobuo, Pandita Tej K. (2017). A multifaceted role for MOF histone modifying factor in genome maintenance. Mechanisms of Ageing and Development.

[36] Gupta A (2005). Involvement of human MOF in ATM function. Mol. Cell. Biol..

[37] Kumar R (2011). Purkinje cell-specific males absent on the first (mMof) gene deletion results in an ataxia-telangiectasia-like neurological phenotype and backward walking in mice. Proc. Natl Acad. Sci. USA..

[38] Gupta A (2014). MOF phosphorylation by ATM regulates 53BP1-mediated double-strand break repair pathway choice. Cell Rep..

[39] Bhadra MP (2012). The role of MOF in the ionizing radiation response is conserved in *Drosophila melanogaster*. Chromosoma.

[40] Gupta A (2008). The mammalian ortholog of *Drosophila* MOF that acetylates histone H4 lysine 16 is essential for embryogenesis and oncogenesis. Mol. Cell. Biol..

[41] Yasuhara T (2018). Human Rad52 promotes XPG-mediated R-loop processing to initiate transcription-associated homologous recombination repair. Cell.

[42] Piazza A, Heyer WD (2019). Homologous Recombination and the Formation of Complex Genomic Rearrangements. Trends Cell Biol..

[43] Refuerzo JS (2015). Liposomes: a nanoscale drug carrying system to prevent indomethacin passage to the fetus in a pregnant mouse model. Am. J. Obstet. Gynecol..

[44] Rodrigue A (2006). Interplay between human DNA repair proteins at a unique double-strand break in vivo. EMBO J..

[45] Kim J (2018). Replication stress shapes a protective chromatin environment across fragile genomic regions. Mol. Cell..

[46] Pandita RK (2006). Mammalian Rad9 plays a role in telomere stability, S- and G2-phase-specific cell survival, and homologous recombinational repair. Mol. Cell. Biol..

[47] Weinstock DM, Nakanishi K, Helgadottir HR, Jasin M (2006). Assaying double-strand break repair pathway choice in mammalian cells using a targeted endonuclease or the RAG recombinase. Methods Enzymol..

