# Supplementary material for: Pre-existing H4K16ac levels in euchromatin drive DNA repair by homologous recombination in S-phase
Source: Commun Biol. 2019 Jul 5;2:253. doi: 10.1038/s42003-019-0498-z (PMC6611875; doi:10.1038/s42003-019-0498-z)
Supplement: Supplementary file 1 — Supplementary Information [file 42003_2019_498_MOESM1_ESM.pdf]

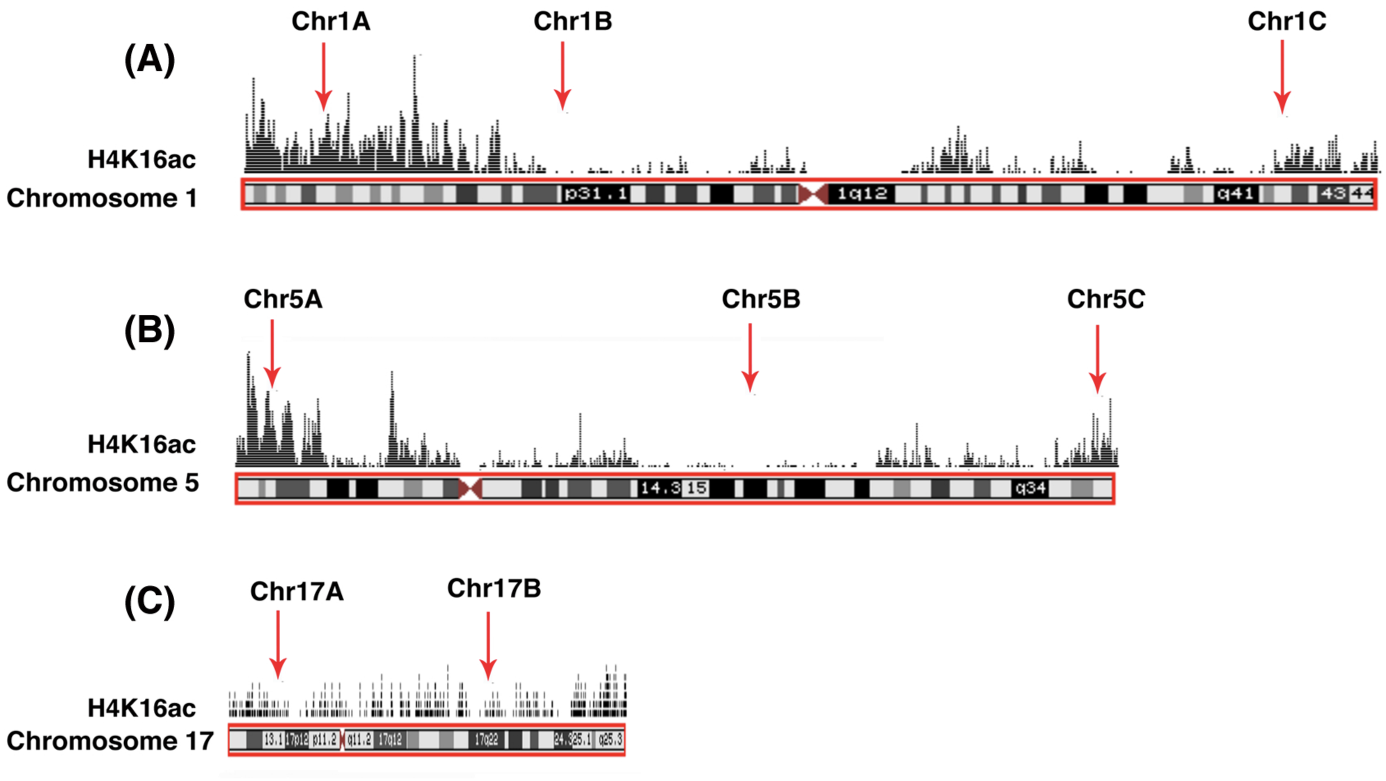

Supplementary Figure 1. H4K16ac distribution on three different chromosomes has been described previously (1).

A) Three sites (Chr1A, Chr1B, Chr1C) are chosen on chromosome 1. B) Three sites (Chr5A, Chr5B, Chr5C) are chosen on chromosome 5. C) Two sites (Chr17A, Chr17B) are chosen on chromosome 17. These sites are chosen for DNA DSB repair.

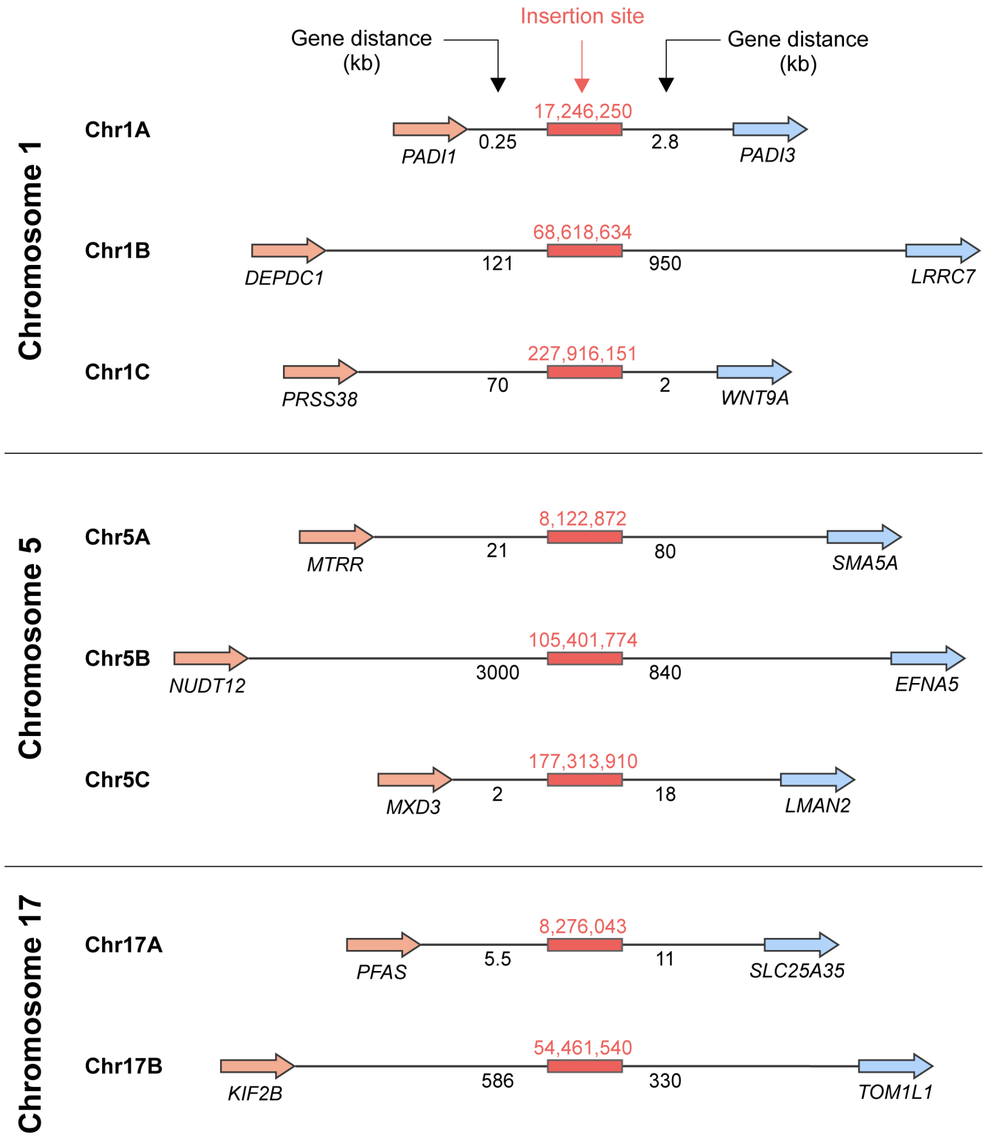

Supplementary Figure 2: Location of insertion sites

The exact location of I-SceI or DR-GFP or EJ-GFP insertion and the distance between the insertion site and near known genes. The gene-rich regions are the sites, which are less that 25 kb from the nearest gene. Primers for PCR/ChIP were designed around the insertion sites.

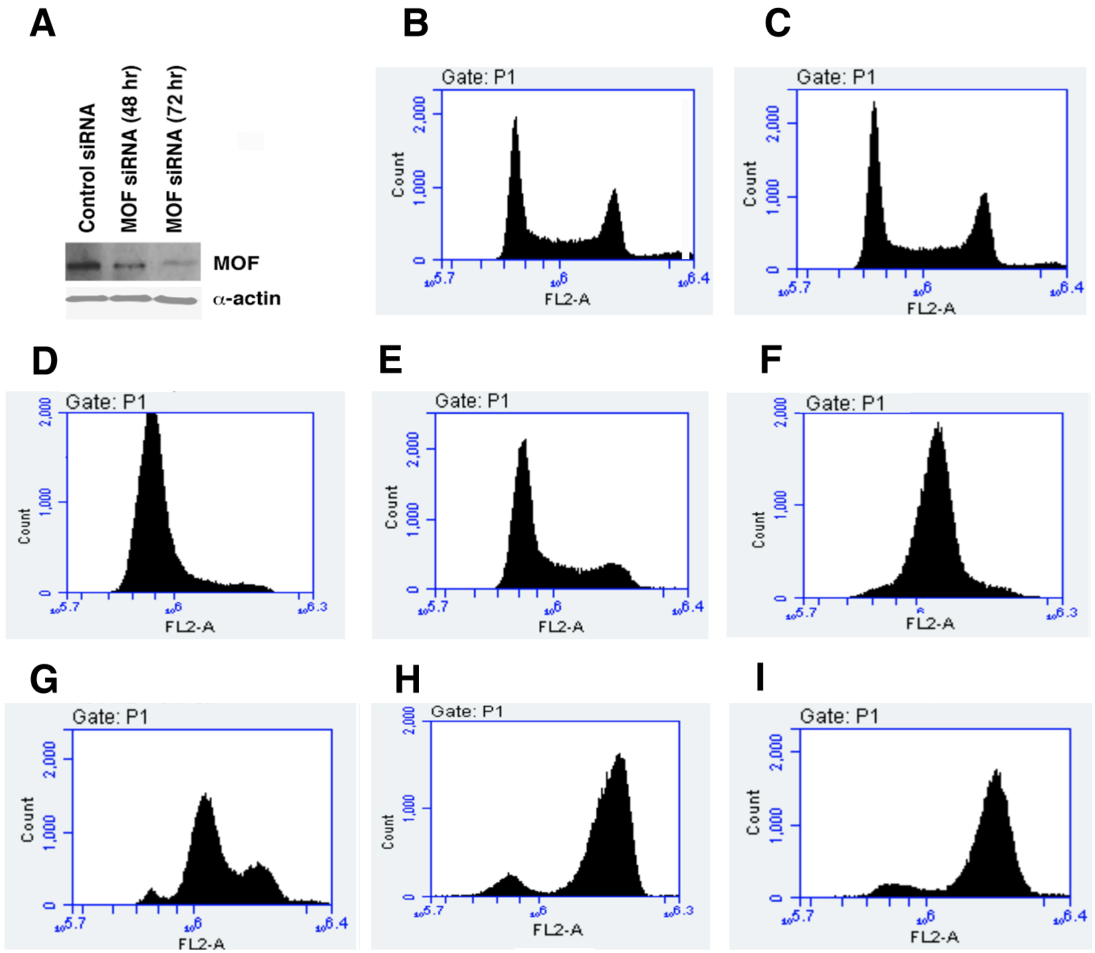

Supplementary Figure 3: Flow data at different time points for cells synchronized as described previously (2) and examined for DNA content to determine the cell cycle stage.
A) Western blot showing the depletion of MOF with specific siRNA from Dharmacon (cat #: D-014800-04-0005) in H1299 cell. B) Exponentially growing untreated cells. C-I) Cells were released for different time periods from a thymidine/aphidicolin block. C) Examined immediately after drug treatment. D) Cells with 99% in G1 phase. E) Cells washed with fresh media and 2 h after G1 block release. F) About 90% cells are in S-phase after 4 h release from G1 block. G) Cells after 7 h release of G1 block. H) About 75% cells in G2 phase after 8 h release of G1 block. I) About 90% cells in G2/M phase after 10 h release of G1 block.

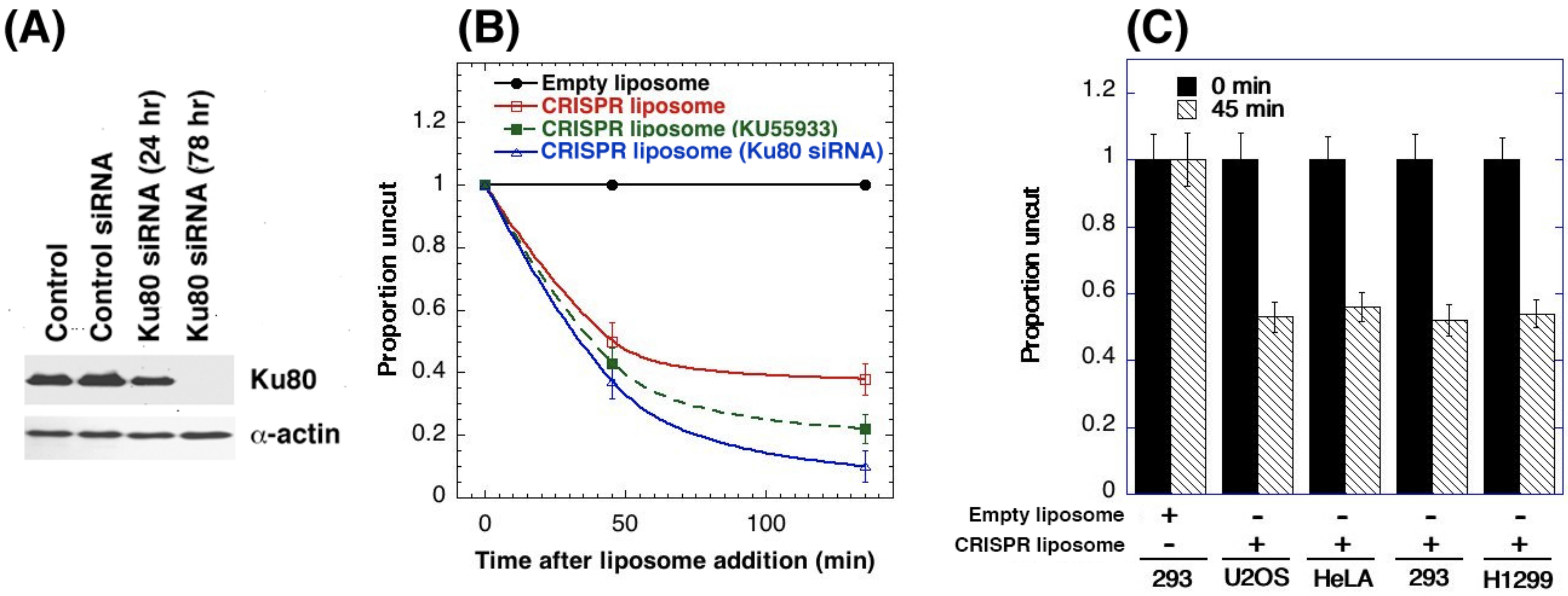

Supplementary Figure 4: DNA cut with the CRISPR liposome at Chr1A.
A) Western blot showing depletion of Ku80 with specific siRNA from Dharmacon (cat #: L-010491-00-0005) in H1299 cells. B) Kinetics of DNA cut with CRISPR liposome at Chr1A in the presence of ATM inhibitor and depletion of Ku80 by specific siRNA. **C**) DNA cut with CRISPR liposome at Chr1A in different cell lines.

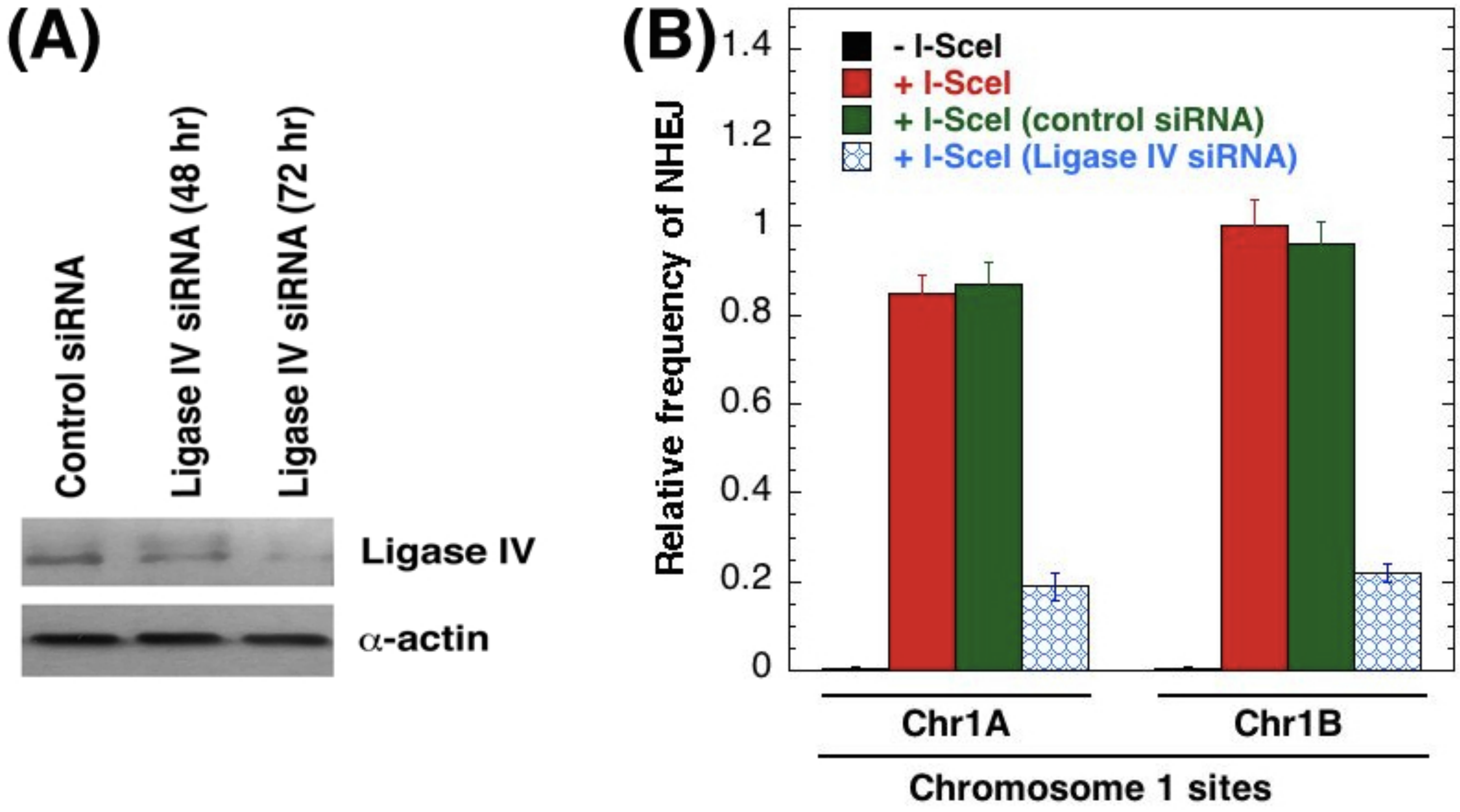

Supplementary Figure 5: Frequency of DSB repair by NHEJ at the different sites.

A) Western blot showing depletion of Ligase IV with specific siRNA from Dharmacon (cat #: L-004254-00-0005) in H1299 cells. B) Comparison of the relative frequency of DSB repair by NHEJ at the different sites on chromosome number 1 (Chr1A and Chr1B) with and without depletion of Ligase IV.

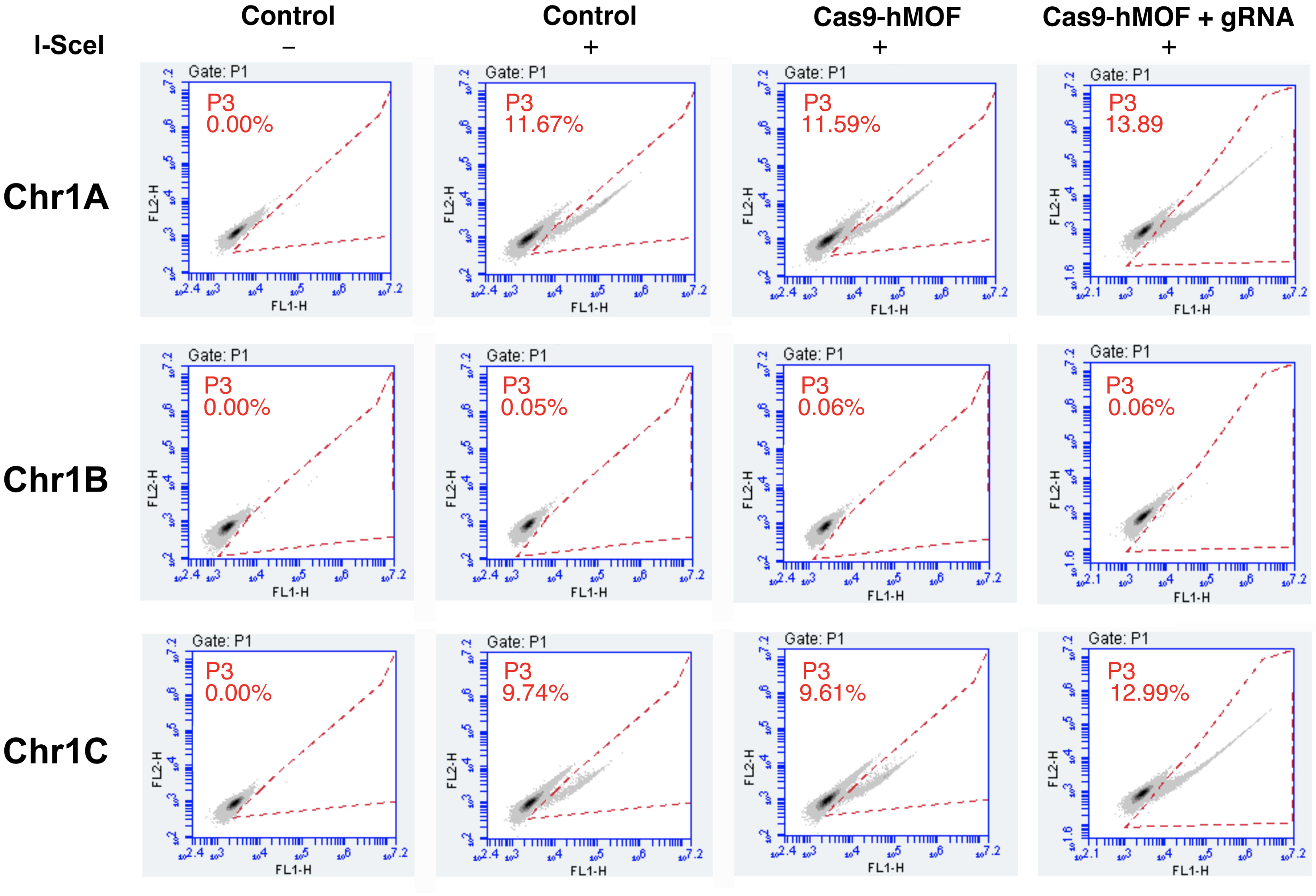

Supplementary Figure 6: Flow analysis of GFP positive cells in order to determine the DSB repair by HR.

Cells with DR-GFP cassettes inserted at the different sites (Chr1A, Chr1B and Chr1C) of chromosome with and without the ectopic expression of Cas9-hMOF and specific gRNA with and without the expression of I-SceI protein.

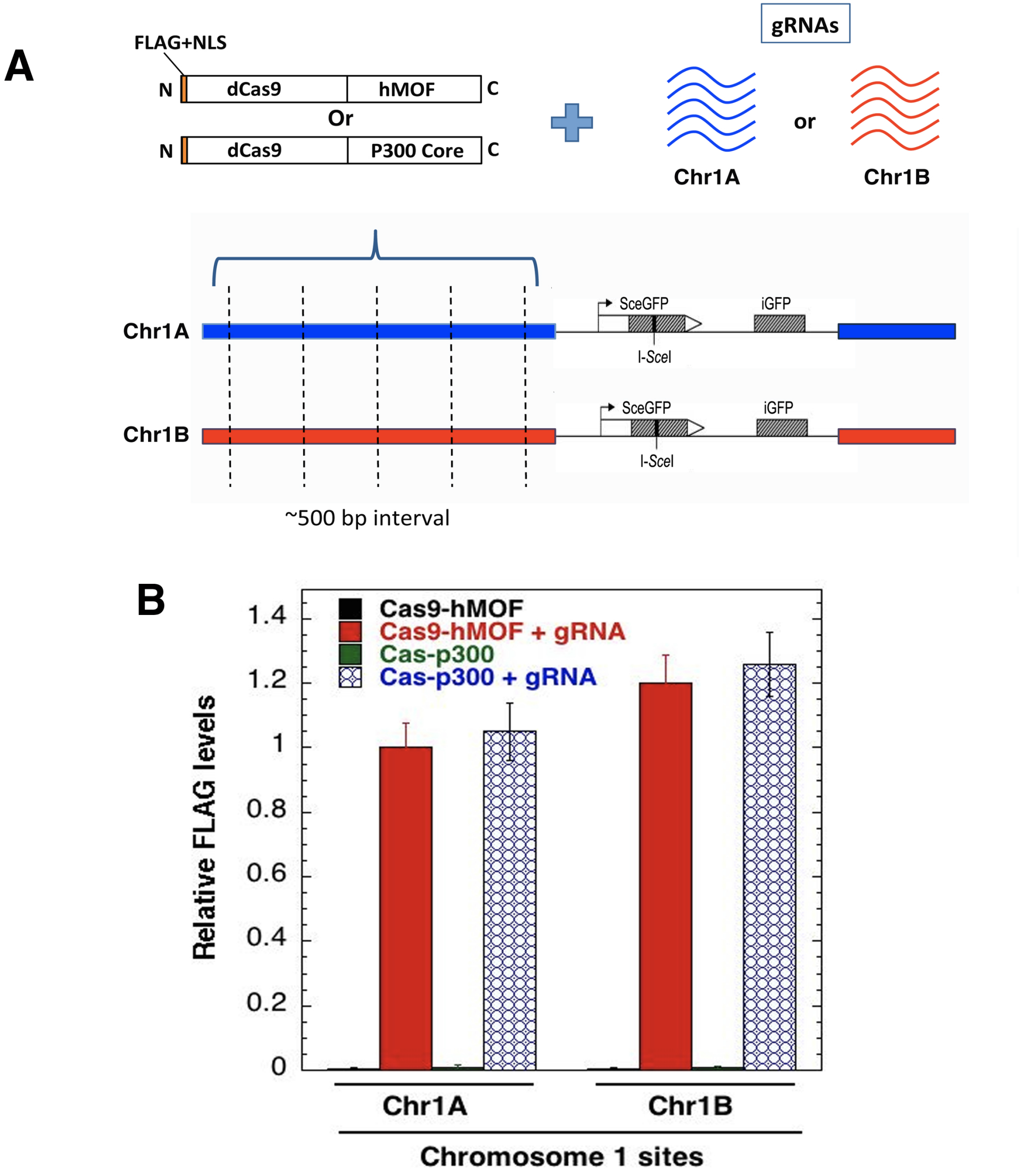

Supplementary Figure 7: Expression of Cas9-hMOF with specific gRNA.
A) Strategy for the expression of site specific Cas9-hMOF and gRNAs. Cas9-hMOF is FLAG tagged.
B) The binding levels of the FLAG tagged hMOF at different sites of DSB on chromosome 1 (Chr1A and Chr1B) analyzed by ChIP/PCR.

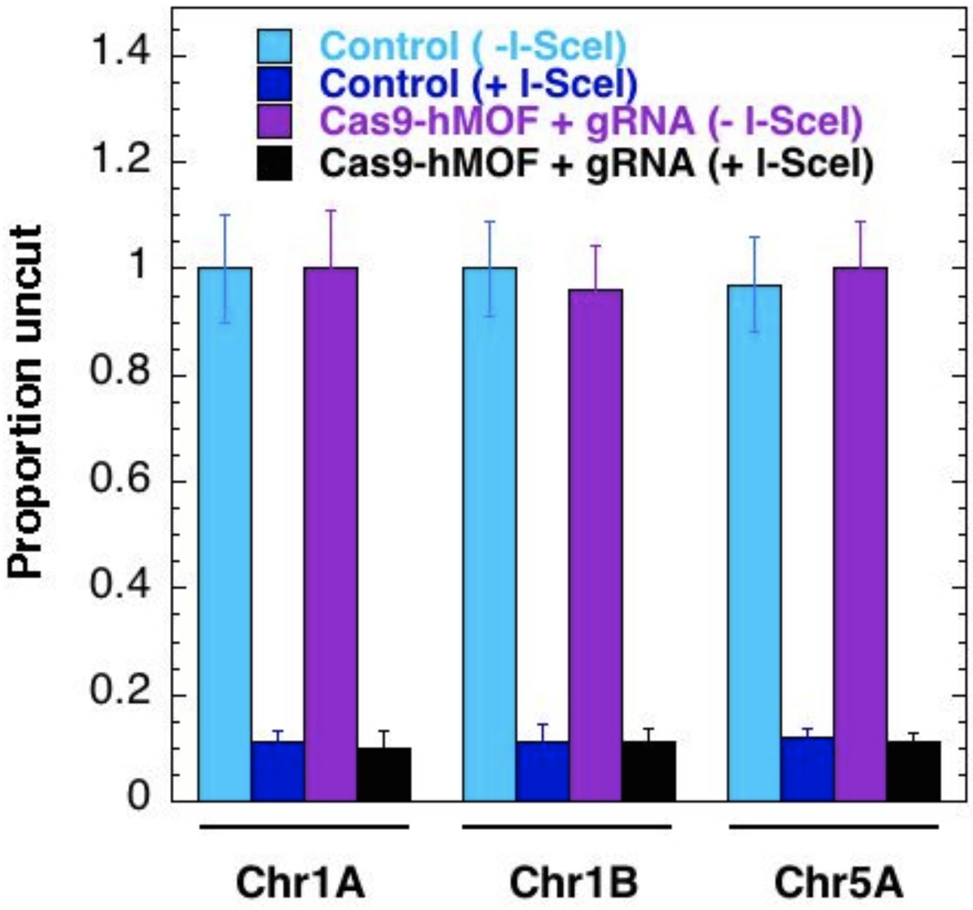

Supplementary Figure 8. Uncut DNA.
Proportion of uncut DNA in at different sites (Chr1A, Chr1B, Chr5A) in cells with and without expression of Cas9-hMOF after 45 min triamcinolone acetonide treatment for I-SceI translocation from cytoplasm to nucleus.

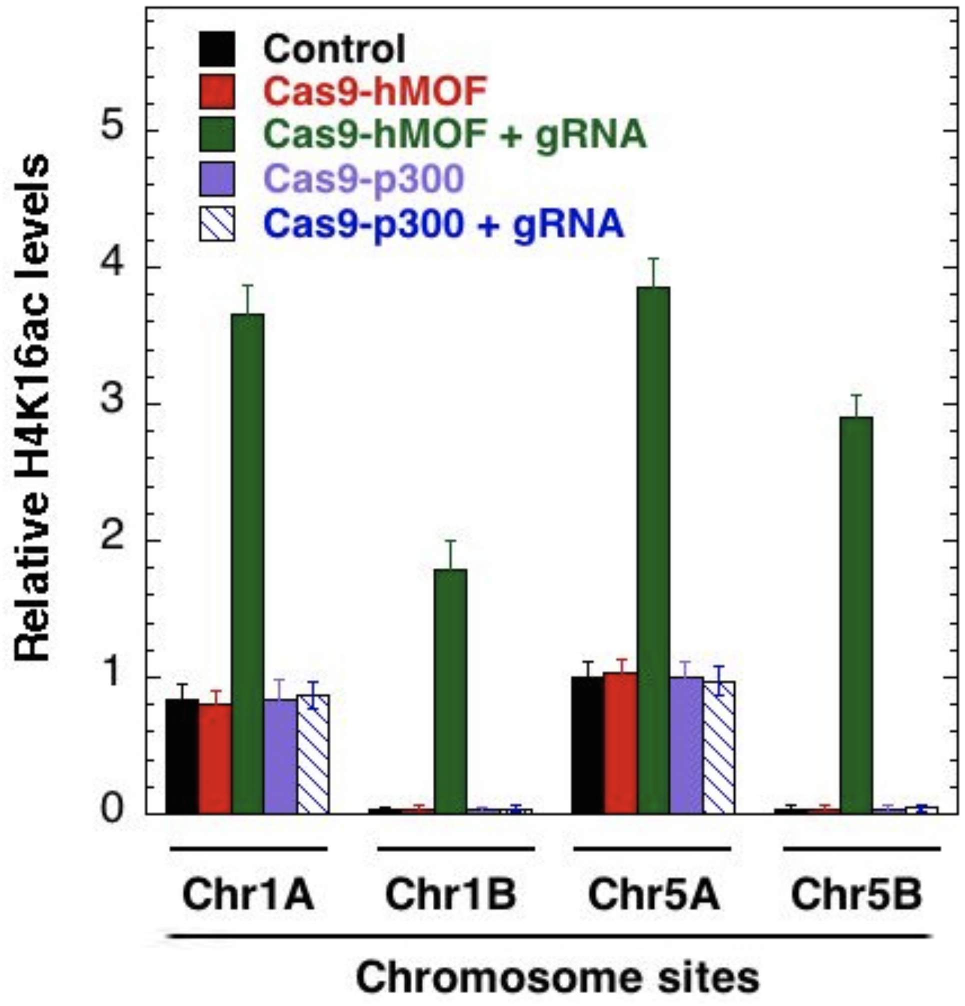

Supplementary Figure 9: Expression of Cas9-hMOF and Cas9-p300. Cells expressing Cas9-hMOF and Cas9-p300 with and without specific gRNA were analyzed for H4K16ac levels by ChIP/PCR at different sites of chromosome (Chr1A, Chr1B, Chr5A, Chr5B).

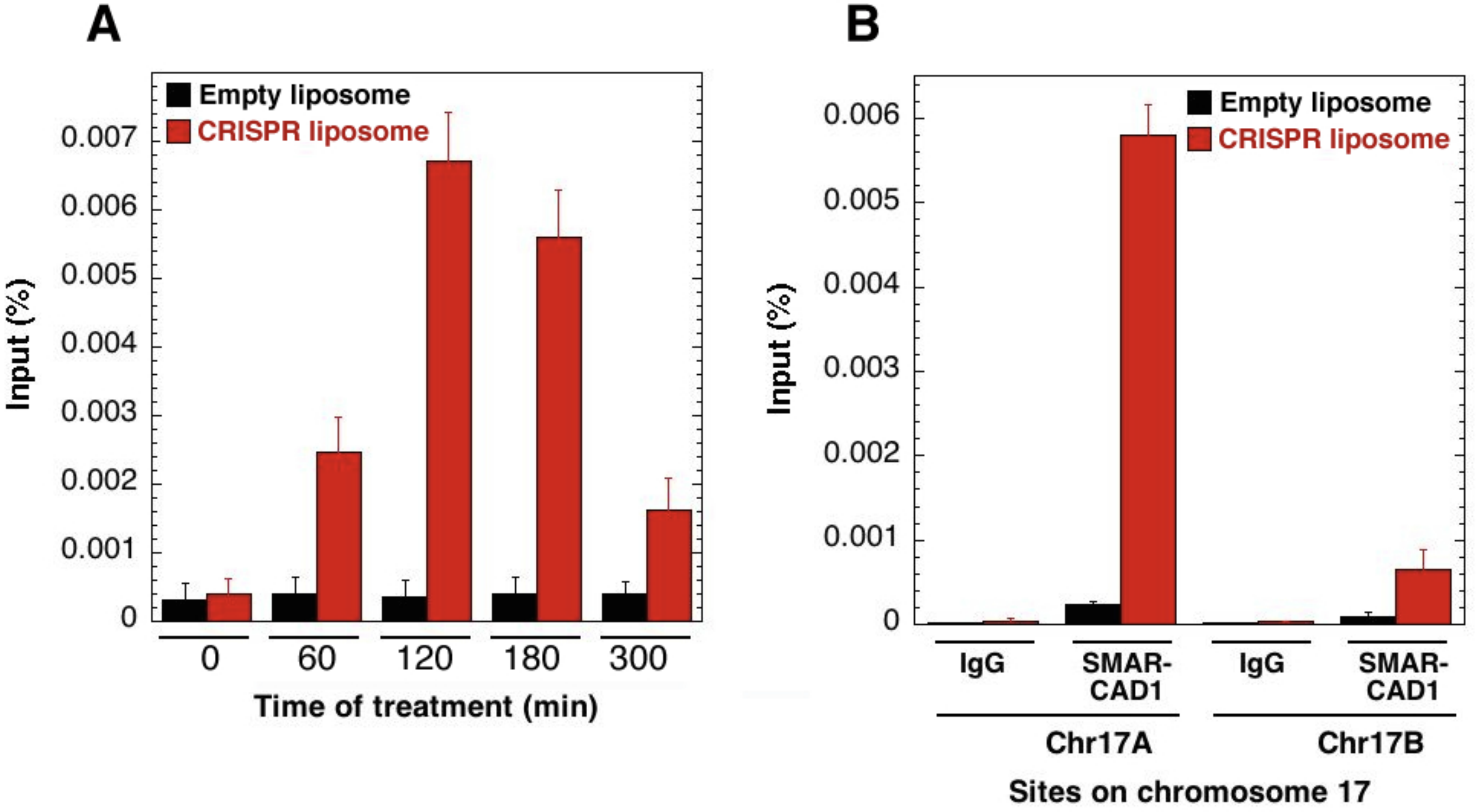

Supplementary Figure 10. SMARCAD1 association with DSB sites with and without induction of DSB with treatment with specific CRISPR liposomes.
Association of SMARACDA1 with DNA DSB site (Chr17A) at different times after the cells were treated with CRISPR liposome for DSB site (Chr17A) of chromosome 17. B) Comparison of SMARCAD1 association at the different sites of chromosome 17 (Chr17A and Chr17B).

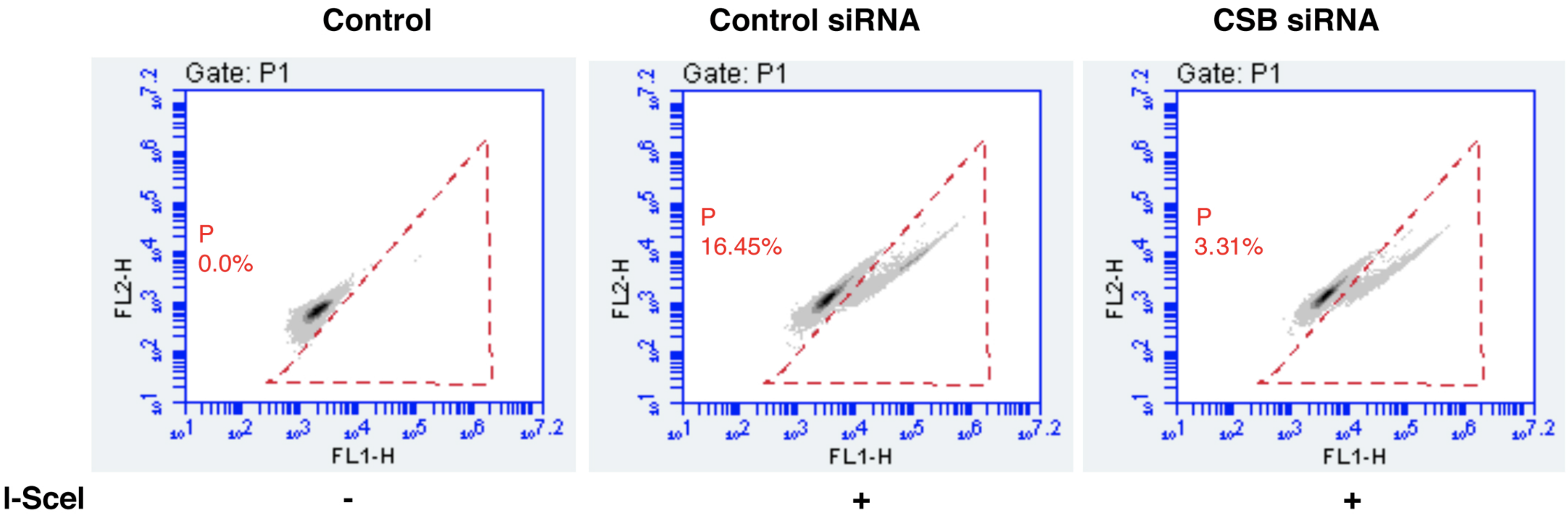

Supplementary Figure 11. Effect of CSB on DSB repair by homologous recombination. Flow analysis of GFP positive cells with DR-GFP cassette (Chr1A) with and without depletion of CSB with specific siRNA after I-SceI expression to induce DSB.

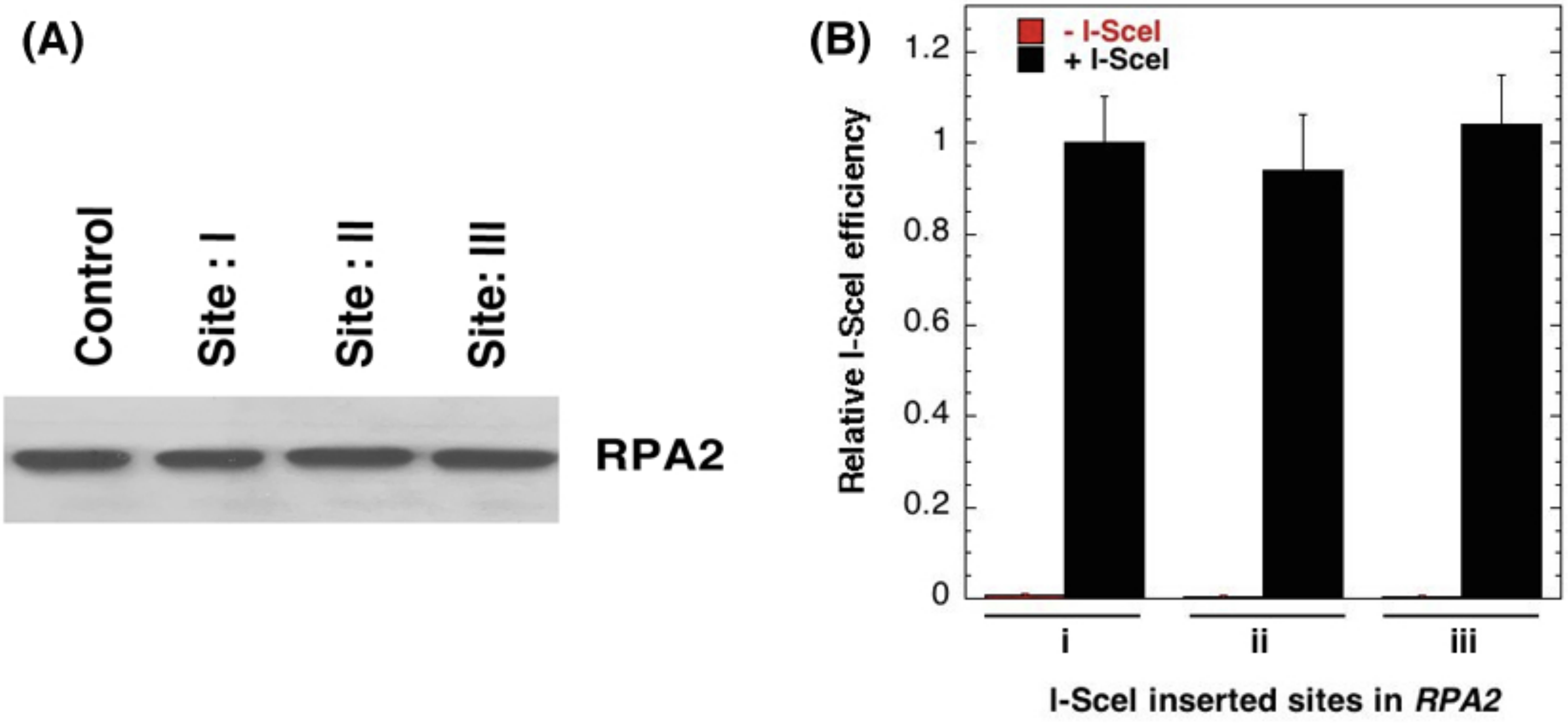

Supplementary Figure 12. RPA2 protein levels and DNA cut.
A) Western blot showing the levels of RPA2 in different clones of I-SceI sites in H1299.
B) Efficiency of DNA cut by I-SceI expression at the three different sites of RPA2 (I, ii, iii) as determined by LM-PCR using the site-specific primers.

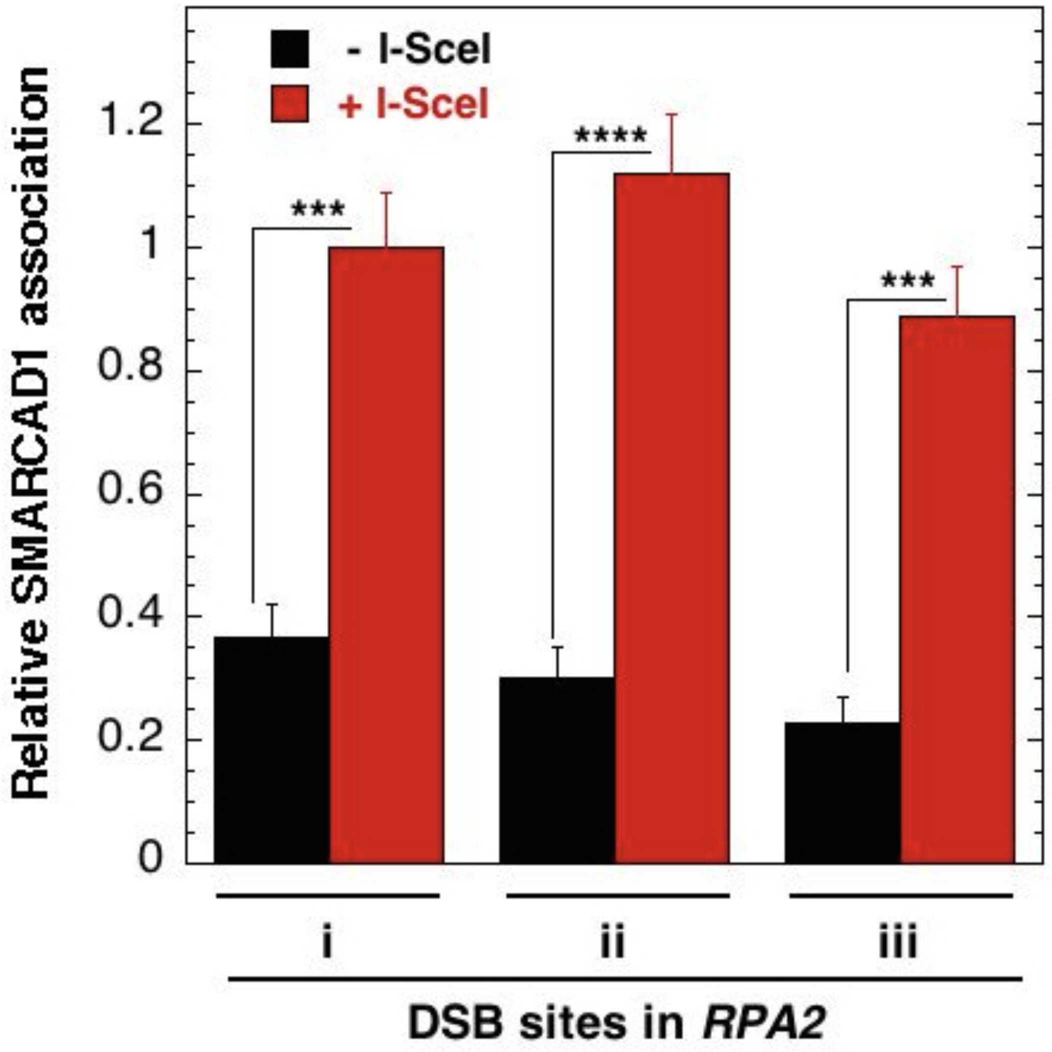

Supplementary Figure 13. Recruitment of SMARCAD1 at DSB.
Association of SMARCAD1 at the different sites of RPA2 gene before and after induction of DSB.

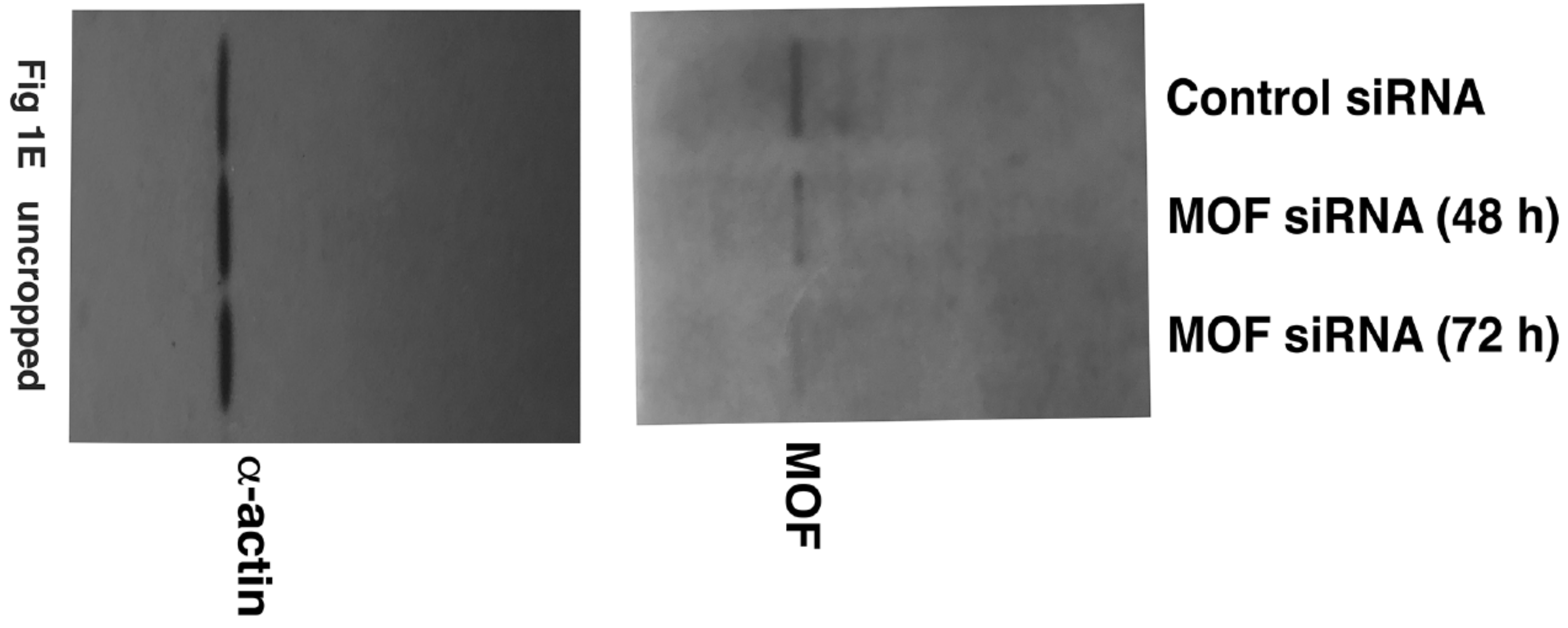

Supplementary Figure 14. Uncropped gel image from main Figure 1e.

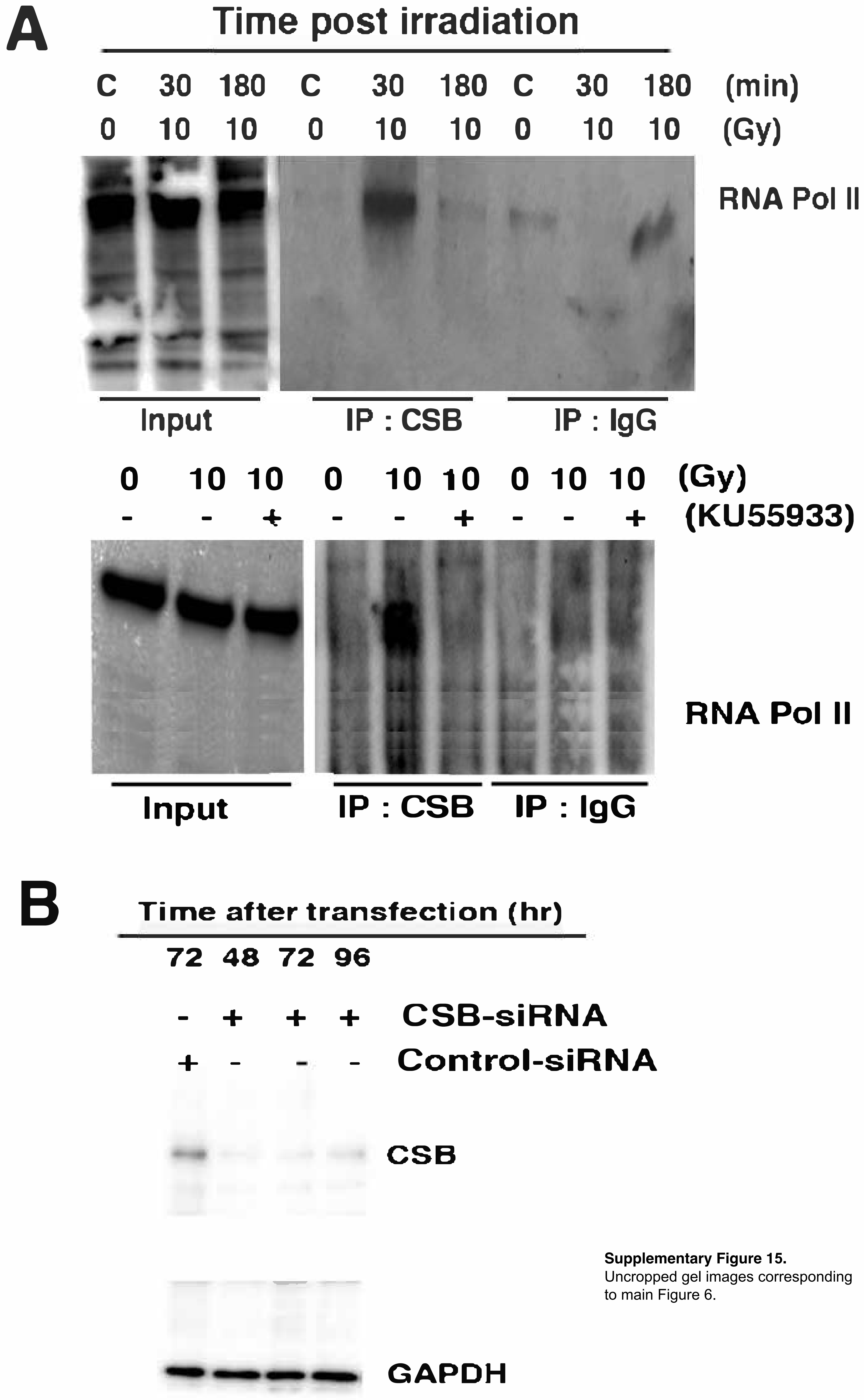

Supplementary Figure 15.
Uncropped gel images corresponding to main Figure 6.

REFERENCES

1. Horikoshi N, Kumar P, Sharma GG, Chen M, Hunt CR, Westover K, et al. Genome-wide distribution of histone H4 Lysine 16 acetylation sites and their relationship to gene expression. Genome Integr. 2013;4(1):3.

2. Rodrigue A, Lafrance M, Gauthier MC, McDonald D, Hendzel M, West SC, et al. Interplay between human DNA repair proteins at a unique double-strand break in vivo. EMBO J. 2006;25(1):222-31.

- ChrlA: 1-SceI, DR-GFP, EJ-GFP
- ChrlB: 1-SceI, DR-GFP, EJ-GFP
- ChrlC: DR-GFP
- ChrSA: 1-SceI, DR-GFP
- ChrSB: DR-GFP
- ChrSC: 1-SceI, DR-GFP
- Chr17A: 1-SceI, DR-GFP
- Chr17B: 1-SceI, DR-GFP
- RPA2i: 1-SceI
- RPA2 ii: 1-SceI
- RPA2iii: 1-SceI

Supplementary Table 1
List of the cell lines with inserted 1-SceI sequence, EJ5-GFP and DR-GFP cassette at the different sites on chromosome 1, 5, and 17.
