# Supplementary material for: Pre-existing H4K16ac levels in euchromatin drive DNA repair by homologous recombination in S-phase
Source: Commun Biol. 2019 Jul 5;2:253. doi: 10.1038/s42003-019-0498-z (PMC6611875; doi:10.1038/s42003-019-0498-z)
Supplement: Supplementary file 2 — Reporting Summary [file 42003_2019_498_MOESM2_ESM.pdf]

nature research

Corresponding author(s): Tej K. Pandita

Last updated by author(s): May 24, 2019

Reporting Summary

Nature Research wishes to improve the reproducibility of the work that we publish. This form provides structure for consistency and transparency in reporting. For further information on Nature Research policies, see Authors & Referees and the Editorial Policy Checklist.

Statistics

For all statistical analyses, confirm that the following items are present in the figure legend, table legend, main text, or Methods section.

n/a	Confirmed	
☐	☒	The exact sample size (n) for each experimental group/condition, given as a discrete number and unit of measurement
☐	☒	A statement on whether measurements were taken from distinct samples or whether the same sample was measured repeatedly
☐	☒	The statistical test(s) used AND whether they are one- or two-sided<br>*Only common tests should be described solely by name; describe more complex techniques in the Methods section.*
☒	☐	A description of all covariates tested
☒	☐	A description of any assumptions or corrections, such as tests of normality and adjustment for multiple comparisons
☐	☒	A full description of the statistical parameters including central tendency (e.g. means) or other basic estimates (e.g. regression coefficient) AND variation (e.g. standard deviation) or associated estimates of uncertainty (e.g. confidence intervals)
☒	☐	For null hypothesis testing, the test statistic (e.g. F, t, r) with confidence intervals, effect sizes, degrees of freedom and P value noted<br>*Give P values as exact values whenever suitable.*
☒	☐	For Bayesian analysis, information on the choice of priors and Markov chain Monte Carlo settings
☒	☐	For hierarchical and complex designs, identification of the appropriate level for tests and full reporting of outcomes
☒	☐	Estimates of effect sizes (e.g. Cohen's d, Pearson's r), indicating how they were calculated

Our web collection on statistics for biologists contains articles on many of the points above.

Software and code

Policy information about availability of computer code

Data collection	The following software programs were used: Photoshop CS6, KaleidaGraph, Excel.<br>No custom made codes were used for this study.
Data analysis	The following software programs wer used: ImageJ/FIJI (NIH), Photoshop CS. No custom made codes were used for this study,

For manuscripts utilizing custom algorithms or software that are central to the research but not yet described in published literature, software must be made available to editors/reviewers. We strongly encourage code deposition in a community repository (e.g. GitHub). See the Nature Research guidelines for submitting code & software for further information.

Data

Policy information about availability of data

All manuscripts must include a data availability statement. This statement should provide the following information, where applicable:

- Accession codes, unique identifiers, or web links for publicly available datasets
- A list of figures that have associated raw data
- A description of any restrictions on data availability

We declare that all the data supporting the findings of this study are available within the paper and the supplementary information files.

Field-specific reporting

Please select the one below that is the best fit for your research. If you are not sure, read the appropriate sections before making your selection.

☒ Life sciences ☐ Behavioural & social sciences ☐ Ecological, evolutionary & environmental sciences

Life sciences study design

All studies must disclose on these points even when the disclosure is negative.

Sample size	The sample size is the number of cells examined and or the number of experimental replicates, as described.
Data exclusions	We did not exclude any data.
Replication	Each experiment was repeated three to four times. All findings were reliably reproduced as noted in the text. All attempts at replication were successful.
Randomization	Samples were randomly allocated to the experiments.
Blinding	There was no blinding of sample in our study.

Reporting for specific materials, systems and methods

We require information from authors about some types of materials, experimental systems and methods used in many studies. Here, indicate whether each material, system or method listed is relevant to your study. If you are not sure if a list item applies to your research, read the appropriate section before selecting a response.

Materials & experimental systems

n/a	Involved in the study	
☐	☒	Antibodies
☐	☒	Eukaryotic cell lines
☐	☐	Palaeontology
☐	☐	Animals and other organisms
☐	☐	Human research participants
☐	☐	Clinical data

Methods

n/a	Involved in the study	
☒	☐	ChIP-seq
☐	☒	Flow cytometry
☒	☐	MRI-based neuroimaging

Antibodies

Antibodies used	histone H4 (Millipore, 05858), H4K16ac (Millipore, 07329) SMARCAD1 (Santa Cruz Biotech, sc-162233, Novus Bio NB100-79835), γ-H2AX (Millipore, JBW301), Ku80 (Cell Signaling, 2180P), RAD51 (Abcam, ab213), BRCA1 (Santa CruzBiotech,sc-642), 53BP1 (Santa Cruz Biotech, sc-22760), CSB (Santa Cruz, 25371, 166042), GAPDH (Protein Tech, HRP-6004), were used for Western blotting, immunoprecipitation and immunofluorescence. Antibodies used for ChIP assay were histone H4 (Millipore, 05858), H4K16ac (Millipore, 07329), SMARCAD1 (Abnova, H00056916-BO1P), γ-H2AX (Millipore, JBW301), 53BP1 (Santa Cruz Biotech, sc-22760), BRCA1 (Abcam), Ku80 (Thermo Fisher,MA5-12933), RAD51(Abcam,ab176458), RAD51 (Abcam, ab176458), RNA polymerase ll (Abcam, ab817)
Validation	Commercial validated reagents were used. No signals were seen in the secondary only control.

Eukaryotic cell lines

Policy information about cell lines

Cell line source(s)	H1299, HEK293, U2OS, HeLa
Authentication	ATCC
Mycoplasma contamination	Negative
Commonly misidentified lines (See ICLAC register)	Not applicable

Palaeontology

Specimen provenance	Not applicable
Specimen deposition	Not applicable

Dating methods	Not applicable

☒ Tick this box to confirm that the raw and calibrated dates are available in the paper or in Supplementary Information.

Animals and other organisms

Policy information about studies involving animals; ARRIVE guidelines recommended for reporting animal research

Laboratory animals	Not applicable
Wild animals	Not applicable
Field-collected samples	Not applicable
Ethics oversight	Not applicable

Note that full information on the approval of the study protocol must also be provided in the manuscript.

Human research participants

Policy information about studies involving human research participants

Population characteristics	Not applicable
Recruitment	Not applicable
Ethics oversight	Not applicable

Note that full information on the approval of the study protocol must also be provided in the manuscript.

Clinical data

Policy information about clinical studies

All manuscripts should comply with the ICMJE guidelines for publication of clinical research and a completed CONSORT checklist must be included with all submissions.

Clinical trial registration	Not applicable
Study protocol	Not applicable
Data collection	Not applicable
Outcomes	Not applicable

Flow Cytometry

Plots

Confirm that:

☒ The axis labels state the marker and fluorochrome used (e.g. CD4-FITC).

☒ The axis scales are clearly visible. Include numbers along axes only for bottom left plot of group (a 'group' is an analysis of identical markers).

☒ All plots are contour plots with outliers or pseudocolor plots.

☒ A numerical value for number of cells or percentage (with statistics) is provided.

Methodology

Sample preparation	Samples prepared according to the manufacture BD Accuri flow cytometry
Instrument	BD Accuri
Software	BD Accuri C6 Software
Cell population abundance	0.02-20% GFP positive
Gating strategy	50,000 cells

☐ Tick this box to confirm that a figure exemplifying the gating strategy is provided in the Supplementary Information.
